# Middle Interlayer Engineered Ferroelectric NAND Flash Overcoming Reliability and Stability Bottlenecks for Next‐Generation High‐Density Storage Systems

**DOI:** 10.1002/advs.202510155

**Published:** 2025-08-23

**Authors:** Giuk Kim, Sangho Lee, Hyojun Choi, Yangjin Jung, Woongjin Kim, Sanghyun Park, Kwangyou Seo, Kwangsoo Kim, Wanki Kim, Daewon Ha, Mincheol Shin, Jinho Ahn, Sanghun Jeon

**Affiliations:** ^1^ Department of Electrical engineering Korea Advanced Institute of Science and Technology (KAIST) Daejeon 34141 South Korea; ^2^ Semiconductor R&D Center Samsung Electronics Hwaseong‐si 18367 South Korea; ^3^ Department of Material Science Engineering Hanyang University Seoul 04763 South Korea

**Keywords:** charge‐trap‐flash, ferroelectrics, memory device, NAND flash, polarization

## Abstract

Multilevel storage and low‐voltage operation position ferroelectric transistors as promising candidates for next‐generation nonvolatile memory. Among them, gate‐injection‐type ferroelectric transistors offer improved vertical scalability and power efficiency for three‐dimensional (3D) NAND flash. However, their intricate interplay between polarization switching and charge trapping complicates systematic understanding of degradation mechanisms, limiting strategies to improve reliability and stability. Here, gate stack engineering incorporating middle interlayers within HfZrO_x_ matrix is presented to modulate polarization dynamics, strengthening the coupling of dual mechanisms and overcoming long‐standing reliability and stability bottlenecks in ferroelectric NAND operation. This approach achieves a memory window up to 11 V, an operating voltage below 18 V, triple‐level‐cell retention beyond 10 years, disturbance immunity, and 54% reduced threshold voltage variability. A 20% reduction in program voltage compared to conventional NAND enables aggressive vertical scaling, leading to 25% higher bit‐density. Furthermore, analytical modeling provides insights into gate stack optimization. These findings establish ferroelectric NAND as a scalable, energy‐efficient solution for next‐generation storage.

## Graphical abstract

A gate‐injection‐type Fe‐NAND architecture is developed by incorporating an Al_2_O_3_ middle interlayer to address key challenges in reliability and scalability. The engineered gate stack enables a wide memory window at low voltage, enhances retention beyond 10 years, suppresses disturbance and threshold voltage variability, and supports array‐level operation, providing a promising pathway for high‐density ferroelectric NAND flash memory.

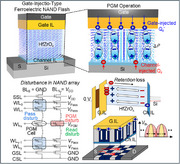

## 1 Introduction

The exponential growth in the big data era has intensified the demand for higher bit‐per‐area efficiency in memory technologies.^[^ ^1^, ^2^ ^]^ To address this, 3D NAND flash memory has pursued aggressive vertical stacking and pitch scaling of word lines (WL) and interline spacers.^[^ ^3^, ^4^ ^]^ However, further increases in bit‐density face physical limitations due to constraints on chip thickness, which restrict additional vertical stacking.^[^ ^5^ ^]^ Consequently, enhancing the number of memory cells per string within a limited height has emerged as a key strategy to sustain bit‐density growth. However, this approach intensifies reliability challenges, as elevated program/erase voltages (*V*_PGM_/*V*_ERS_) required for multilevel operation exacerbate breakdown issues in ultrathin spacers.^[^ ^6^ *^‐^* ^8^ ^]^ Therefore, a new memory architecture capable of achieving a wide memory window (MW) with multilevel capability at reduced operating voltages is urgently required.

Hafnia‐based ferroelectric transistors have garnered considerable attention for their complementary metal‐oxide‐semiconductor (CMOS) compatibility, multilevel data storage capability, and low‐power operation.^[^ ^9^, ^10^ ^]^ To harness these properties in high‐density nonvolatile memory, various innovative gate structures have been explored to realize next‐generation ferroelectric NAND (Fe‐NAND) flash.^[^ ^11^, ^12^, ^13^, ^14^, ^15^, ^16^, ^17^, ^18^, ^19^, ^20^ ^]^ For instance, floating‐gate ferroelectric transistors have demonstrated large MWs at reduced operating voltages.^[^ ^15^, ^16^, ^17^ ^]^ However, these devices require gate stack thicknesses exceeding 50 nm, significantly larger than that of state‐of‐the‐art charge‐trap flash (CTF) memory (≈20 nm), thereby increasing lateral footprint and limiting scalability in 3D NAND. Moreover, physically isolating the floating‐gate in 3D configurations complicates fabrication and elevates costs. Alternatively, double‐gate ferroelectric transistors offer enhanced performance at lower voltages, but encounter similar integration challenges in vertical NAND due to process complexity.^[^ ^18^, ^19^, ^20^ ^]^ Therefore, refining gate structures to maintain ≈20 nm total gate stack thickness while ensuring compatibility with existing 3D NAND fabrication processes is essential.

**Figure 1:**
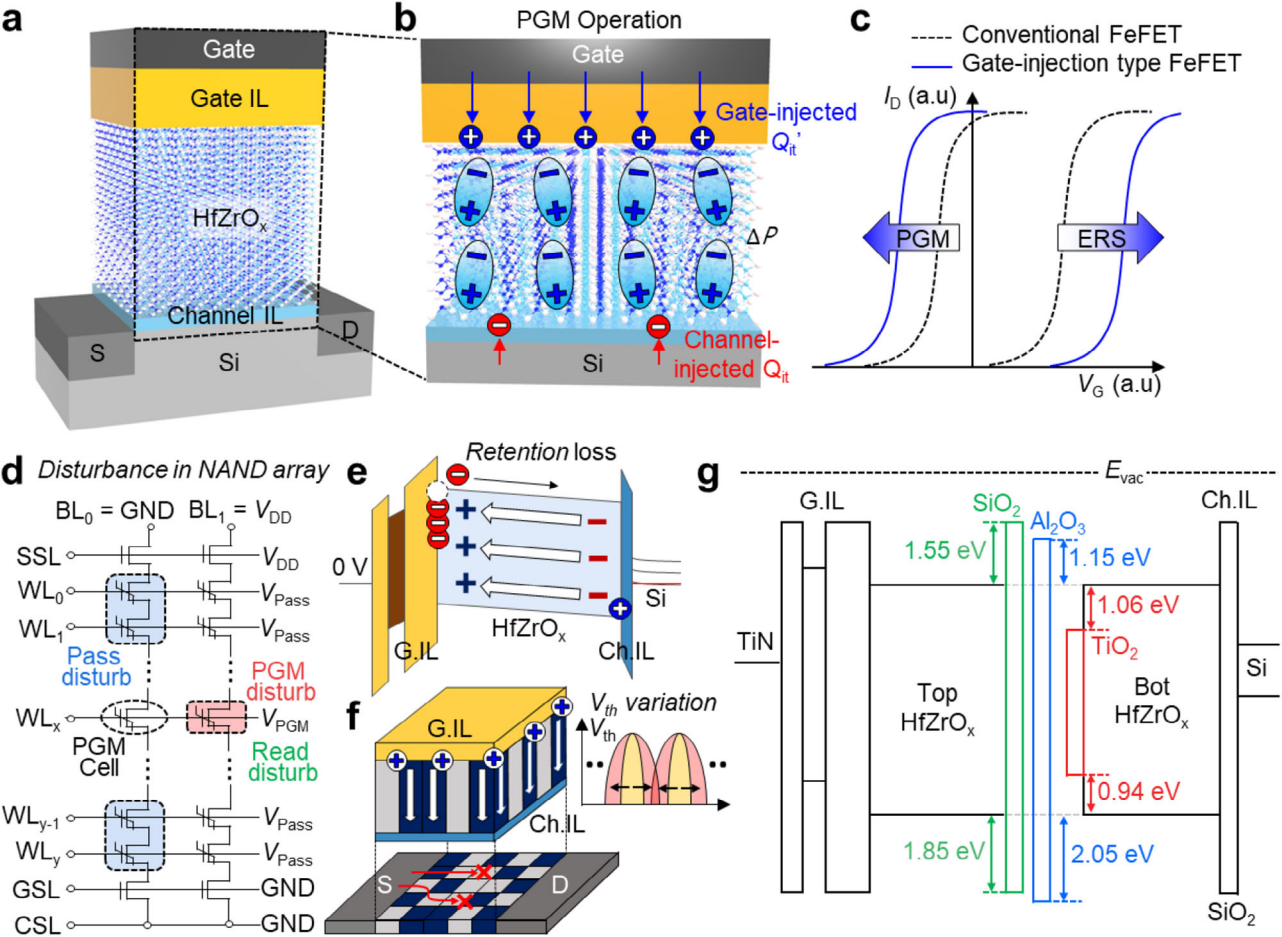
Key challenges of gate‐injection type ferroelectric NAND (Fe‐NAND) flash. a) Device structure of a Fe‐NAND unit cell featuring a metal‐gate interlayer (gate IL)–ferroelectrics–channel interlayer (channel IL)–semiconductor (MIFIS) gate stack. b) Charge formation mechanisms during program (PGM) operation, illustrating gate‐injected interface‐trap charges (*Q*_it_’) between gate IL/HfZrO_x_, polarization switching in HfZrO_x_, and channel‐injected interface‐trap charges (*Q*_it_) between channel IL/HfZrO_x_. c) Enlarged memory window (MW) enabled by coupled polarization and charge trapping in gate‐injection‐type ferroelectric FET (FeFET). Key challenges for reliable Fe‐NAND array operation include: d) lack of gate stack strategies to mitigate PGM, pass, and read disturbance; e) insufficient understanding of retention degradation mechanisms; and f) unresolved threshold voltage (*V*_th_) variability induced by ferroelectric domain and charge trapping effects. g) Energy band diagrams of Fe‐NAND gate stacks with various middle interlayers (mid‐ILs) employed in this study to address these issues.

Ferroelectric transistors featuring a metal‐gate interlayer (gate IL)–ferroelectrics–channel interlayer (channel IL)–semiconductor (MIFIS) gate stack utilize two distinct dynamics of polarization switching and charge trapping for memory operations (**Figure** **1**A). During programming, polarization switching is accompanied by tunneling‐induced charge injection from the gate, forming interface‐trapped charges (*Q*_it_’) at the gate IL/HfZrO_x_ interface (Figure 1B). The synergy of dual effects lowers the operating voltages and expands the MW (Figure 1C). Additionally, the total gate stack thickness of MIFIS transistors is comparable to conventional CTF devices, ensuring 3D integration compatibility without increasing the lateral footprint. Nevertheless, prior studies have mainly focused on enhancing individual device characteristics, with limited attention to array‐level performance optimization.^[^ ^21^, ^22^, ^23^, ^24^, ^25^, ^26^, ^27^, ^28^ ^]^ Furthermore, the complex interaction between polarization switching and charge trapping has obscured the fundamental understanding of disturbance phenomena (Figure 1D) and long‐term reliability degradation (Figure 1E). The compounded threshold voltage (*V*_th_) variability stemming from the dual mechanisms also remains a key challenge (Figure 1F).

In this work, we extend gate‐injection type ferroelectric transistors to Fe‐NAND flash technology through gate stack engineering, building on earlier insights and further enhancing array‐level performance via detailed analysis of reliability and integration metrics.^[^ ^24^ ^]^ Specifically, we introduce a middle‐interlayer (mid‐IL) within the ferroelectric layer of the MIFIS gate stack (Figure 1G). The optimized mid‐IL modulates phase composition and domain configurations in the HfZrO_x_ film, enhancing polarization characteristics, as verified through material analysis and 3D phase‐field simulations. Furthermore, proper mid‐IL integration maintains the coupling between polarization and gate‐injected *Q*_it_’ under retention and disturbance conditions, leading to unprecedented performance improvements. Analytical modeling and novel measurements support these findings. The optimized ferroelectric transistors feature a 24 nm‐thick total gate stack, ensuring compatibility with high‐density 3D integration. They exhibit an expanded MW (>10 V) while operating at reduced *V*_PGM_/*V*_ERS_ (18/−18 V), alongside exceptional retention performance (>10 years) and complete immunity to disturbance effects, surpassing previously reported ferroelectric transistor performances. In addition, device simulations confirm that mid‐IL insertion stabilizes channel conductance fluctuations arising from the polycrystalline and polymorphic nature of HfZrO_x_, reducing *V*_th_ variability by 54%. Scaling analysis suggests that the 20% reduction in *V*_PGM_ enables aggressive spacer pitch scaling, allowing a 25% increase in bit‐density compared to conventional charge‐trap based NAND. In summary, we investigate a gate‐injection‐type MIFIS FeFET structure with a mid‐IL embedded within the HfZrO_x_ matrix, and for the first time, identify the underlying mechanisms of reliability degradation, *V*_th_ variability, and disturbance. Whereas prior works that primarily focused on improving isolated characteristics, our approach systematically analyzes the interplay among key reliability factors and demonstrates how mid‐IL engineering contributes to overall device performance. We experimentally validate that the inserted mid‐IL not only improves electrical metrics such as MW and retention but also effectively suppresses V_th_ variation arising from the polymorphic and polycrystalline nature of HfZrO_x_. Furthermore, we demonstrate, for the first time, the feasibility of program inhibit operation using global self‐boosting, a critical functionality for 3D Fe‐NAND integration. These results collectively highlight the novelty and practicality of our approach, offering a robust and application‐driven solution to overcoming key limitations of gate‐injection‐type Fe‐NAND architectures.

## 2 Results

### 2.1 Mid‐IL Engineering of Polarization Dynamics in HfZrO_x_

**Figure 2:**
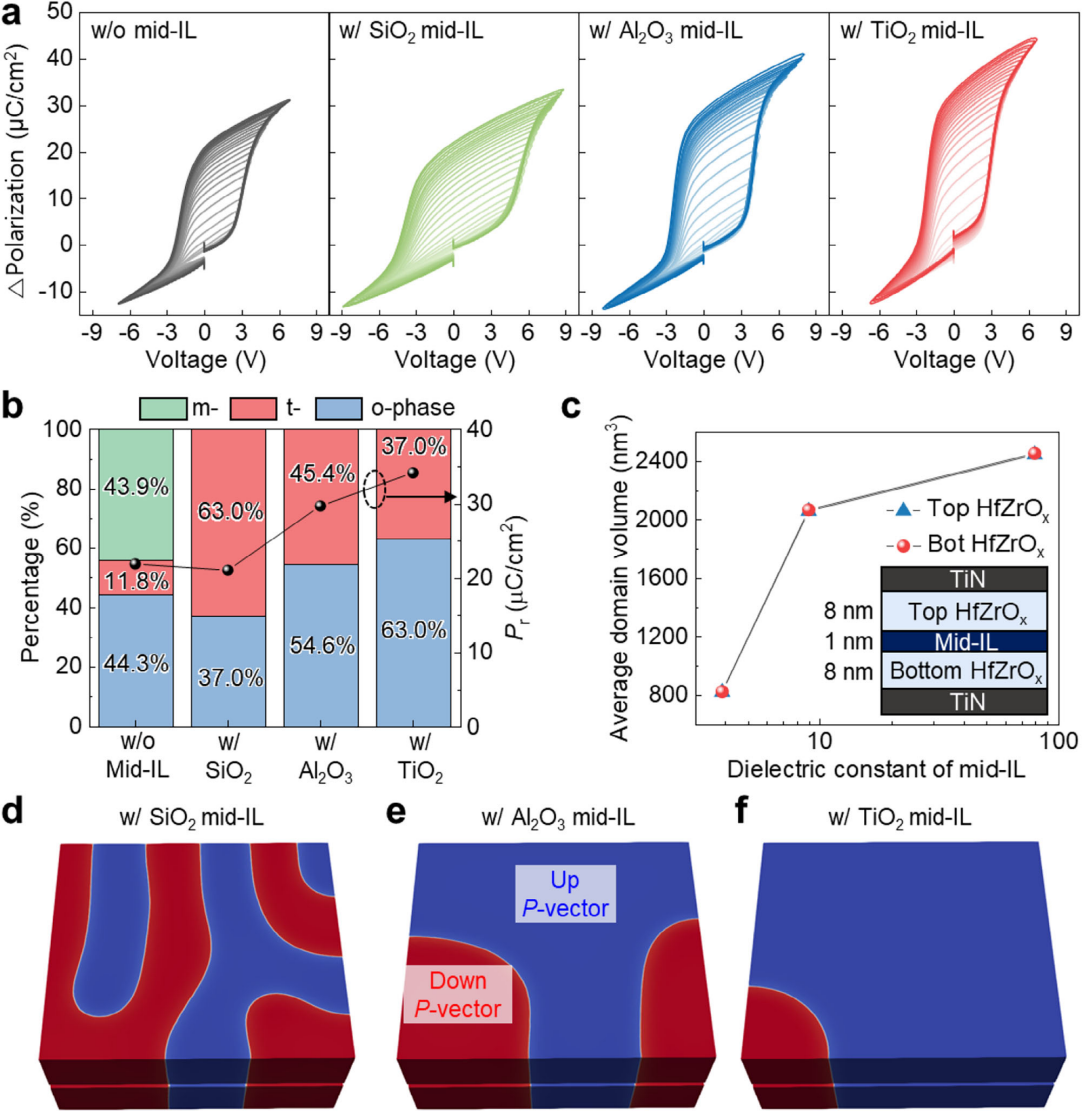
Effect of mid‐IL on polarization dynamics and domain configuration in HfZrO_x_. a) Polarization–voltage (*P*–*V*) and subloop characteristics of HfZrO_x_ films with various mid‐ILs, highlighting variations in remnant polarization (*P*_r_) and partial switching behavior. b) Phase composition from grazing‐incidence X‐ray diffraction (GIXRD) (left y‐axis) and corresponding *P*_r_ values (right y‐axis) for each mid‐IL. c) Average domain volume in top and bottom HfZrO_x_ layers as a function of mid‐IL dielectric constant, extracted from 3D phase‐field simulations. Inset shows the simulated TiN/HfZrO_x_/mid‐IL/HfZrO_x_/TiN stack. Simulated domain configurations in HfZrO_x_ films with d) SiO_2_, e) Al_2_O_3_, and f) TiO_2_ mid‐ILs, illustrating the effect of mid‐IL selection on domain configuration.

The integration of mid‐ILs within HfZrO_x_ film offers a precise approach to tailoring polarization dynamics for ferroelectric transistors. Embedded mid‐ILs physically constrain continuous grain growth, thereby thermodynamically influencing phase distribution.^[^ ^29^, ^30^, ^31^ ^]^ Moreover, the intrinsic properties of mid‐ILs induce lattice strain in adjacent ferroelectric layers, modulating phase transition kinetics.^[^ ^32^ ^]^ Furthermore, mid‐IL incorporation alters the internal free energy landscape, effectively affecting domain configurations.^[^ ^33^, ^34^ ^]^ To systematically examine these effects, we introduced 1‐nm‐thick SiO_2_, Al_2_O_3_, and TiO_2_ layers into the HfZrO_x_ matrix (Figure S1, Supporting Information). Polarization–voltage (*P*–*V*) measurements revealed clear variations in coercive voltage (*V*_C_), remnant polarization (*P*_r_), and subloop behavior, underscoring the crucial role of mid‐IL selection in modulating ferroelectric properties (**Figure** **2**A). Specifically, *V*_C_ increases as the dielectric constant of the mid‐IL decreases. This trend originates from voltage redistribution across the gate stack, where lower dielectric constant mid‐ILs allocate a larger fraction of the applied voltage to the ferroelectric layer, necessitating higher switching voltage.

In contrast, *P*_r_ increases with higher dielectric constant mid‐ILs, indicating enhanced stabilization of the orthorhombic (o‐) phase in HfZrO_x_. Grazing‐incidence X‐ray diffraction (GIXRD) analysis further confirmed the phase composition changes driven by mid‐IL integration (Figure S2, Supporting Information). In 17‐nm‐thick HfZrO_x_ without a mid‐IL, the monoclinic (m‐) phase dominates, while mid‐ILs suppress m‐phase formation (Figure 2B). This effect is attributed to the mid‐IL's role in restricting grain growth, modulating the thermodynamic nucleation process.^[^ ^29^, ^30^, ^31^ ^]^ The evolution of o‐ and tetragonal (t‐) phases is strongly influenced by mid‐IL selection: from SiO_2_ to Al_2_O_3_ to TiO_2_, t‐phase formation is progressively suppressed, whereas o‐phase stabilization is promoted. These phase variations correlate with the coefficient of thermal expansion (CTE) of the mid‐ILs, which increases from 0.5 × 10^−6^ /K (SiO_2_) to 4.2 × 10^−6^ /K (Al_2_O_3_) and 9.9 × 10^−6^ /K (TiO_2_). ^[^ ^35^, ^36^, ^37^ ^]^ During post‐annealing cooling process, higher CTE generates greater tensile stress in adjacent HfZrO_x_ layers, kinetically favoring o‐phase stabilization.^[^ ^32^, ^37^ ^]^

Additionally, increasing the dielectric constant of mid‐IL induces an abrupt polarization subloop response under applied voltages (Figure S3, Supporting Information), where polarization switching remains inactive at low voltages but occurs sharply beyond a threshold. This behavior is attributed to increased domain volume within top and bottom HfZrO_x_ films (Figure 2C). The total free energy of hafnia‐based ferroelectrics is described using the time‐dependent Ginzburg–Landau (TDGL) equation:

(1) $$-\rho \frac{\partial P}{\partial t}=\frac{\partial }{\partial P}\int dV\left(f_{\mathrm{bulk}}+f_{\mathrm{grad}}+f_{\mathrm{elec}}\right)$$

where *P* is polarization, *f*_bulk_ is the bulk free energy density, *f*_grad_ is the gradient free energy density, and *f*_elec_ is the electrostatic free energy density. The total free energy of HfZrO_x_ is governed by three key components: *f*_bulk_, associated with spontaneous polarization; *f*_grad_, related to multi‐domain formation; and *f*_elec_, linked to electrostatic potential. Particularly, the balance between *f*_grad_ and *f*_elec_ determines the multi‐domain configuration.^[^ ^33^ ^]^ A lower dielectric constant in the mid‐IL strengthens the depolarization field within HfZrO_x_, thereby increasing *f*_elec_. To counterbalance, the system elevates *f*_grad_, leading to higher domain wall energy and greater spatial polarization variations. As a result, the domain volume decreases, yielding a denser domain configuration. These trends were visually confirmed by 3D phase‐field simulations for HfZrO_x_ films incorporating different mid‐ILs as shown in Figure 2D–F. Details of the simulation framework are provided in Figure S4 (Supporting Information). In summary, the intrinsic material properties of mid‐ILs enable precise modulating of *V*_C_, *P*_r_, and subloop behavior in HfZrO_x_ films. This capability facilitates optimized polarization dynamics in ferroelectric field‐effect transistors (FeFETs), enhancing memory performance.

### 2.2 Enhanced Memory Performance through Mid‐IL Integration

Continuous scaling of WLs and spacers is essential for advancing high‐density NAND flash memory. To meet this goal, unit cell devices must support multilevel operation at *V*_PGM_ below 18 V while achieving a minimum MW of 7 V for triple‐level‐cell (TLC) operation.^[^ ^7^, ^28^ ^]^ Gate‐injection type ferroelectric transistors can satisfy these stringent requirements by utilizing the cooperative interaction between polarization switching and gate‐injected *Q*_it_’. During program/erase (PGM/ERS) operations, *Q*_it_’ formation facilitates bound charge compensation, which promotes polarization switching. This, in turn, increases band bending at the gate IL, enhancing further *Q*_it_’ formation and establishing a positive feedback loop.^[^ ^22^, ^38^ ^]^ Meanwhile, incremental step pulse programming (ISPP) is the standard method for precise *V*_th_ tuning in NAND flash. However, repeated PGM/read cycles degrade reliability, making high ISPP efficiency a key design goal.^[^ ^39^ ^]^ In conventional charge‐trap NAND cells, which rely solely on charge trapping, the ISPP slope is inherently limited to 1.^[^ ^40^ *^,^* ^41^ ^]^ In contrast, gate‐injection‐type ferroelectric transistors surpass this limitation by leveraging the feedback effects between polarization and *Q*_it_’, offering superior PGM efficiency.

**Figure 3:**
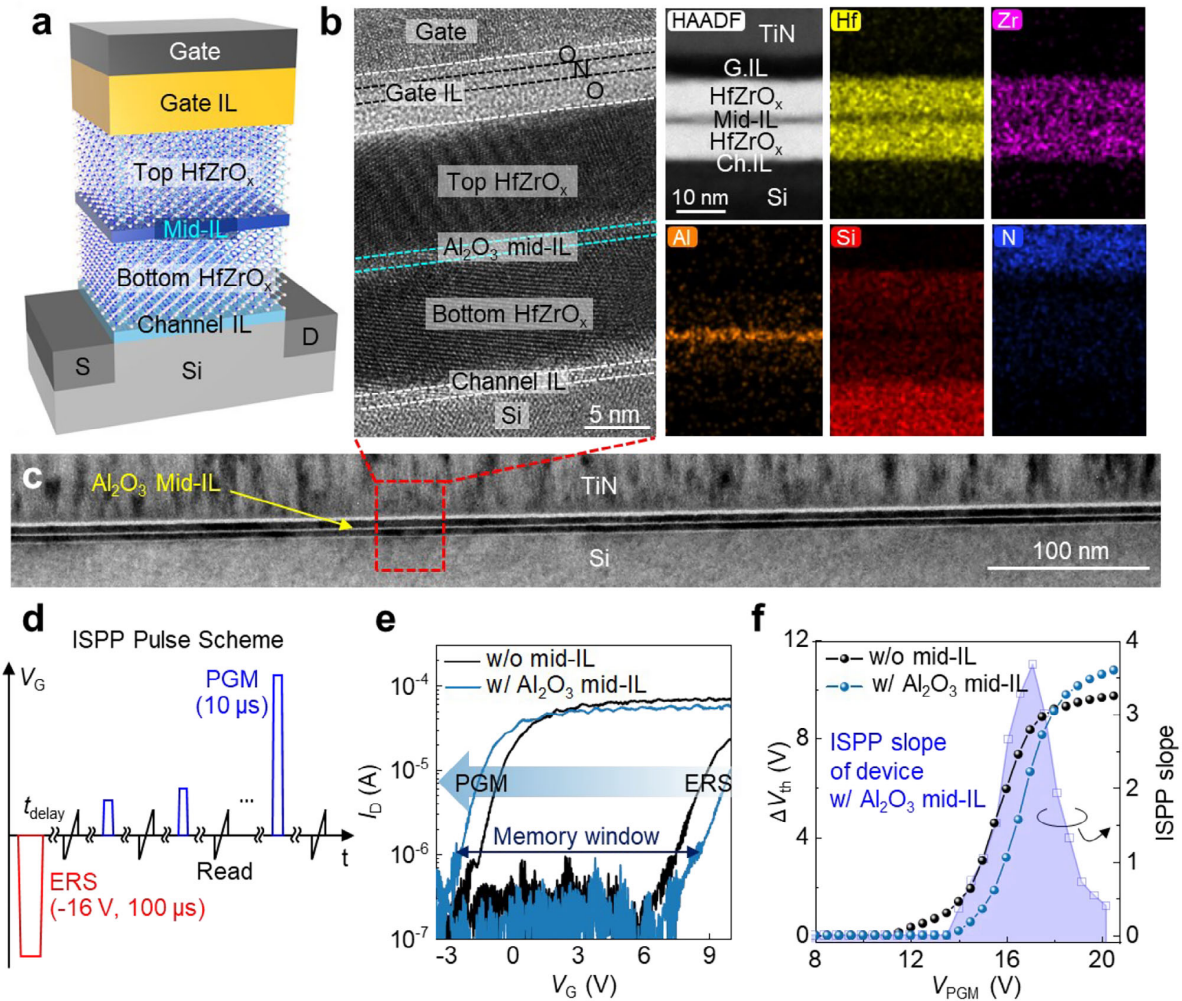
Structural and electrical characterization of mid‐IL‐engineered Fe‐NAND cells. a) Schematic of the Fe‐NAND gate stack incorporating a mid‐IL. b) Cross‐sectional high‐resolution transmission electron microscopy (HRTEM) and energy‐dispersive spectroscopy (EDS) images of the Fe‐NAND unit cell device with an Al_2_O_3_ mid‐IL. c) Low‐magnification TEM image confirming uniform thickness and conformal coverage of the gate stack. d) Incremental step pulse programming (ISPP) scheme used to avoid misestimation from read‐after‐write delay (RAWD) effects. e) Pulse *I*_D_–*V*_G_ characteristics of ferroelectric transistors with and without an Al_2_O_3_ mid‐IL. *V*_th_ is extracted at *I*_D_ = 100 nA × (channel width/length = 100 µm/10 µm). f) ISPP performance comparison, showing Δ*V*_th_ (left y‐axis) and ISPP slope (right y‐axis) as a function of program voltage (*V*_PGM_) for devices with and without an Al_2_O_3_ mid‐IL.

To investigate the impact of mid‐IL integration on polarization dynamics and its interaction with gate‐injected *Q*_it_’, we fabricated MIFIS ferroelectric transistors with various mid‐ILs and evaluated their MW, operating voltages, and ISPP efficiency (**Figure** **3**A). All the mid‐IL candidates investigated in this study, including SiO_2_, Al_2_O_3_, and TiO_2_, are chemically stable without inter‐diffusion into the adjacent HfZrO_x_ layers even under a thermal budget of 600 °C.^[^ ^28^, ^29^, ^37^ ^]^ In particular, lower‐magnification TEM further verifies that the ALD‐based CMOS compatible gate stack achieves uniform thickness and conformal coverage of the Al_2_O_3_ mid‐IL, thereby ensuring compatibility with 3D NAND fabrication processes (Figure 3C). The MW, *V*_PGM_, and ISPP performance of the fabricated transistors were evaluated using the ISPP method. *V*_PGM_ was incrementally increased in 0.5 V steps from a fully erased state with 10‐µs pulse width, while monitoring *V*_th_ evolution (Figure 3D). ISPP operation, which involves a series of short pulses (typically tens of microseconds) with gradually increasing amplitudes, is widely adopted in current NAND flash technology for precise *V*_th_ control of each memory cell.^[^ ^39^, ^42^, ^43^ ^]^ In this study, ISPP measurements were conducted using pulse conditions consistent with those employed in modern 3D NAND architectures. Notably, while conventional CTF devices typically require high‐voltage (−20 – −25 V) and millisecond‐range pulses for erase operations, gate‐injection‐type ferroelectric transistors enable erase functionality at −16 V with 100 µs‐pulses, thereby achieving faster operation and lower power consumption.^[^ ^44^, ^45^ ^]^

The MW properties of ferroelectric transistors with and without an Al_2_O_3_ mid‐IL are shown in Figure 3E, while comparative analyses of other mid‐IL devices are provided in Figure S5 (Supporting Information). Integration of SiO_2_ and Al_2_O_3_ mid‐ILs consistently enhances MW, whereas TiO_2_ mid‐IL unexpectedly degrades MW. Since MW is directly proportional to *V*_C_, the observed improvements with SiO_2_ and Al_2_O_3_ mid‐ILs are attributed to their ability to increase *V*_C_ of HfZrO_x_ films.^[^ ^8^, ^15^, ^16^ ^]^ In particular, the Al_2_O_3_ mid‐IL not only increases *V*_C_ but also improves *P*_r_, resulting in the largest MW among the tested devices. In addition, it has been reported that, during PGM/ERS operations, interface trapped charges injected from the channel side and located between the mid‐IL and HfZrO_x_ layers can undergo flipping induced by polarization switching, and this dynamic behavior contributes to the enhancement of the MW.^[^ ^28^ ^]^ Conversely, despite exhibiting the highest *P*_r_, the TiO_2_ mid‐IL yields the lowest MW. This counterintuitive outcome indicates that additional factors exist beyond the previously reported correlation, wherein higher *P*_r_ induces larger MW in MIFIS ferroelectric transistors.^[^ ^8^, ^46^ ^]^ To further investigate the MW degradation, we analyzed the energy band diagrams of MIFIS gate stacks with and without the TiO_2_ mid‐IL during PGM operations (Figure S6, Supporting Information). Unlike devices without a mid‐IL, the high dielectric constant (≈80) of TiO_2_ introduces a potential well between HfZrO_x_ layers. In fact, TiO_2_ has a relatively low bandgap and high electron affinity, making it prone to forming a potential well. Based on this property, it has been widely utilized as part of the charge trap layer in the previous studies.^[^ ^22^, ^47^, ^48^ ^]^ During PGM/ERS operations, charges injected from the channel become trapped in this potential well, forming *Q*_well_.^[22]^ This *Q*_well_ inhibits polarization switching in adjacent ferroelectric layers, thereby limiting MW expansion. Charge‐sheet based MW modeling corroborates that *Q*_well_ formation directly contributes to MW degradation in devices with TiO_2_ mid‐IL (Figure S7, Supporting Information). To support this interpretation, we analyzed the effective voltage applied across the Ch.IL in MIFIS structures incorporating different mid‐IL materials. Specifically, a constant pulse width of 100 µs was applied while *V*_PGM_ was individually tuned for each device to reach the fully programmed state. The corresponding effective voltages across the Ch.IL (*V*_Ch.IL_), calculated by considering differences in equivalent oxide thickness (EOT), were as follows: for the SiO_2_ mid‐IL, *V*_PGM_ = 18 V and *V*_Ch.IL_ = 1.94 V; for the Al_2_O_3_ mid‐IL, *V*_PGM_ = 16 V and *V*_Ch.IL_ = 1.73 V; for the TiO_2_ mid‐IL, *V*_PGM_ = 19 V and *V*_Ch.IL_ = 2.04 V; and for the device without a mid‐IL, *V*_PGM_ = 15 V and *V*_Ch.IL_ = 1.71 V. These results indicate that the device with the TiO_2_ mid‐IL experiences the highest effective voltage across the Ch.IL, implying enhanced electron injection from the channel‐side and subsequent trapping within the TiO_2_ layer. This analysis, grounded in experimentally measured programming voltages, provides supporting evidence for the proposed *Q*_well_ effect. These findings indicate that *Q*_well_ formation induced by high‐κ mid‐ILs is a key factor constraining MW, emphasizing the need for careful mid‐IL material selection to mitigate detrimental charge trapping effects in Fe‐NAND architectures.

Although the Al_2_O_3_ mid‐IL slightly increases *V*_PGM_ over devices without a mid‐IL, it achieves a Δ*V*_th_ of 9 V at *V*_PGM_ = 18 V, marking a 20% reduction from charge‐trap NAND cells (Figure 3F). In contrast, the SiO_2_ mid‐IL, owing to its low dielectric constant, increases *V*_PGM_ beyond 20 V (Figure S8, Supporting Information). The TiO_2_ mid‐IL also raises the operating voltage, which is attributed to the *Q*_well_ formation inhibiting polarization switching and disrupting the reinforcing feedback between gate‐injected *Q*_it_’ and polarization. The measured ISPP slope further highlights the impact of mid‐IL selection on PGM efficiency. Ferroelectric transistors with an Al_2_O_3_ mid‐IL exhibit a maximum ISPP slope of 3.68, compared to 2.80 for devices without a mid‐IL, indicating a ≈31% improvement in PGM efficiency. This enhancement is closely associated with the high *P*_r_ and abrupt partial polarization switching behavior of HfZrO_x_ films with Al_2_O_3_ mid‐IL (Figure 1A; Figure S3, Supporting Information).^[^ ^7^, ^22^ ^]^ Conversely, SiO_2_ and TiO_2_ mid‐ILs result in degraded ISPP slopes of 2.60 and 0.87, respectively. For SiO_2_ mid‐IL, the reduction in ISPP slope arises from its low dielectric constant, which limits voltage distribution across the gate IL and ferroelectric layers. In the case of TiO_2_ mid‐IL, *Q*_well_ formation suppresses polarization switching and weakens the feedback loop, impairing PGM efficiency. In conclusion, incorporating Al_2_O_3_ mid‐IL significantly improves polarization characteristics, enabling a maximum MW of 11 V and achieving an unprecedented ISPP efficiency of 3.68 in ferroelectric transistors. Furthermore, the reduced *V*_PGM_ of 18 V supports further pitch scaling of WLs and spacers, facilitating seamless integration into 3D NAND architecture. These advancements suggest Fe‐NAND flash can deliver a 25% increase in bit‐density over conventional charge‐trap‐based NAND, highlighting its promise for next‐generation memory applications.

### 2.3 Retention Enhancement via Mid‐IL Integration

Retention is a fundamental requirement for nonvolatile memory, ensuring that stored data remain intact over extended periods without power. In NAND flash memory, retention characteristics govern long‐term data integrity and directly impact system reliability, establishing them as a crucial performance metric for storage applications.^[^ ^49^ ^]^ Despite its significance, the retention loss mechanisms in gate‐injection‐type ferroelectric transistors remain incompletely understood. Here, we elucidate a previously unidentified retention degradation pathway by employing gate engineering with mid‐IL integration and propose a gate stack design strategy to enhance reliability characteristics.

**Figure 4:**
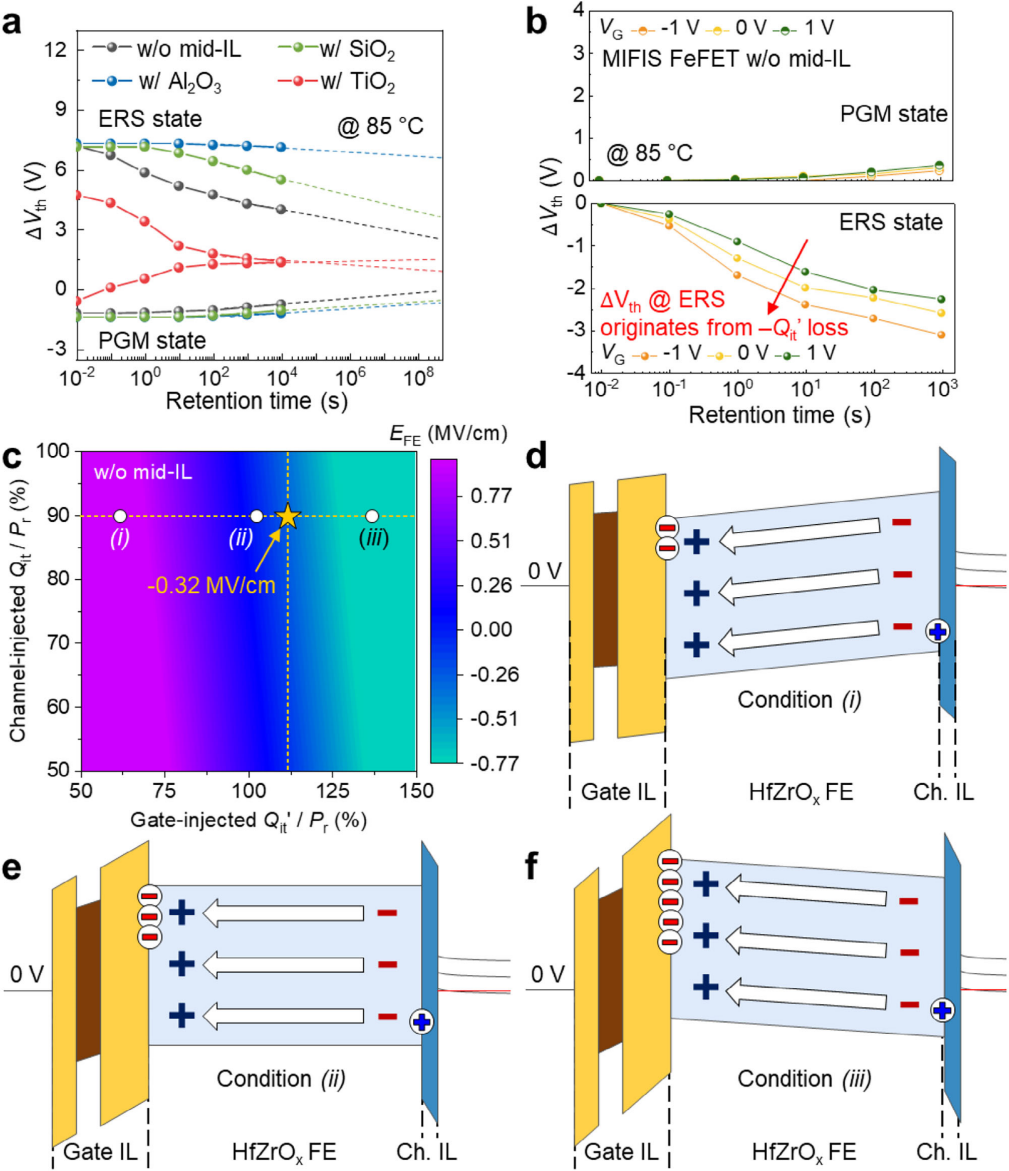
Retention degradation mechanisms in Fe‐NAND devices. a) Retention characteristics of ferroelectric transistors with and without mid‐ILs at 85 °C, showing dependence on mid‐IL type. b) Gate‐biased retention measurements for a ferroelectric transistor without a mid‐IL, revealing the impact of gate bias on charge loss and *V*_th_ shift. c) Contour map of the electric field (*E*_FE_) across the ferroelectric layer in the erase (ERS) state during retention, as a function of (gate‐injected *Q*_it_’)/*P*_r_ and (channel‐injected *Q*_it_)/*P*_r_. Three regimes (*i – iii*) correspond to positive, zero, and negative *E*_FE_ conditions, assuming 90% compensation of *P*_r_ by channel‐injected *Q*_it_. The condition corresponding to an MW of 9 V is denoted by a yellow star. Simulated energy band diagrams of ferroelectric transistor without a mid‐IL, illustrating the energy band configurations under conditions d (*i*), e (*ii*), and f (*iii*), respectively.

The retention characteristics of ferroelectric transistors with various mid‐ILs were evaluated at 85 °C (**Figure** **4**A). The device without a mid‐IL exhibited significant short‐term retention loss in the ERS state (i.e., high *V*_th_ state), consistent with previous studies.^[^ ^21^, ^25^, ^26^, ^27^, ^50^ ^]^ In contrast, the PGM state (i.e., low *V*_th_ state) showed relatively stable retention, with minor Δ*V*_th_ over thousands of seconds. The introduction of a TiO_2_ mid‐IL further degraded retention by promoting *Q*_well_ detrapping, which disrupted polarization charge compensation and accelerated gate‐injected *Q*_it_’ loss. Conversely, SiO_2_ and Al_2_O_3_ mid‐ILs effectively mitigated retention degradation. The SiO_2_ layer reduced short‐term retention loss in the ERS state, although long‐term degradation persisted. Noticeably, the Al_2_O_3_ layer significantly suppressed retention loss over the entire retention period.

Retention degradation is typically more pronounced in the ERS state, where gate‐injected *Q*_it_’ are predominantly electrons, than in the PGM state, where they are primarily holes. Previous studies have suggested that such retention degradation is associated with the detrapping and subsequent escape of gate‐injected *Q*_it_’ through the gate IL.^[^ ^21^, ^23^, ^24^, ^25^, ^26^ ^]^ However, the exact loss pathway of *Q*_it_’ remains experimentally unverified, specifically whether the charge escapes toward the gate‐side or the channel‐side. To clarify the dominant charge loss direction, we applied both positive and negative gate biases during retention measurements. In MIFIS transistors without a mid‐IL, applying a negative gate bias notably increased ∆V_th_ in the ERS state. This indicates that negative bias promotes *Q*_it_’ (i.e., electrons) loss toward the channel‐side by enhancing band bending (Figure S9, Supporting Information). In contrast, a positive gate bias suppressed charge loss toward the channel‐side, thereby reducing Δ*V*th. The PGM state exhibited a similar trend: *Q*_it_’ (i.e., holes) loss toward the channel‐side was mitigated under the opposite bias, resulting in a smaller Δ*V*_th_. On the other hand, due to the inherently lower injection efficiency of holes compared to electrons, the overall Δ*V*_th_ in the PGM state remained relatively small.^[^ ^7^, ^22^ ^]^ Regardless of mid‐IL type, all ferroelectric transistors exhibited the same retention loss behavior (Figure S10, Supporting Information), confirming that charge loss predominantly occurs toward the channel‐side rather than the gate‐side. Consistent with Figure 4A, devices with SiO_2_ and Al_2_O_3_ mid‐ILs exhibited improved retention, while TiO_2_ mid‐IL further worsened retention degradation. The gate‐biased retention measurements do not fully capture all side effects, such as cycling‐induced degradation. Nevertheless, analyzing the physical behavior of trapped charge escape under controlled gate bias conditions during retention has been widely employed in the study of conventional CTF devices.^[^ ^45^, ^51^ ^]^ In this study, to objectively compare the impact of mid‐IL on retention characteristics across devices, we applied a uniform delay time (*t*_delay_) of 100 ms to all measurements, ensuring sufficient detrapping and minimizing *V*_th_ error caused by read‐after‐write delay (RAWD).^[^ ^52^, ^53^ ^]^ In addition, relatively small gate biases of −2, −1, 0, 1, and 2 V were applied during retention to suppress further charge injection, unintended polarization switching, and stress‐induced degradation.

To further investigate the impact of SiO_2_ and Al_2_O_3_ mid‐ILs on retention characteristics, we conducted additional modeling studies (Figure S11, Supporting Information). In the ERS state of MIFIS gate stacks, the effective electric field across the ferroelectric layers (*E*_FE_) was quantified by considering the combined contributions from *P*_r_, gate‐injected *Q*_it_’ and channel‐injected *Q*_it_. Here, the channel‐injected *Q*_it_ refers to the interface‐trapped charge formed between the ferroelectric layer and channel IL during PGM/ERS operations. *E*_FE_ serves as a key parameter, directly reflecting band bending within the HfZrO_x_ layers and providing critical insights into charge stability and retention behavior. For the device without a mid‐IL, a contour plot of *E*_FE_ as a function of (channel‐injected *Q*_it_)/*P*_r_ and (gate‐injected *Q*_it_’)/*P*_r_ is shown in Figure 4C. *E*_FE_ was calculated based on the *P*_r_ value obtained from the polarization dynamics described in Figure 2A. To systematically compare the energy band diagrams, the (channel‐injected *Q*_it_)/*P*_r_ ratio was set to 90%, based on previous studies showing that channel‐injected *Q*_it_ compensated ≈90% of *P*_r_ after a delay time (≈100 ms). ^[^ ^52^, ^53^, ^54^ ^]^

The corresponding energy band diagrams for conditions (*i*), (*ii*), and (*iii*) are illustrated in Figure 4D–F. In condition (*i*), where *E*_FE_ is positive, gate‐injected *Q*_it_’ is relatively small compared to *P*_r_, resulting in band bending that suppresses the *Q*_it_’ loss toward the channel‐side (Figure 4D). In condition (*ii*), where *E*_FE_ is zero, the ferroelectric layer exhibits a flat energy band profile (Figure 4E). Conversely, in condition (*iii*), where *Q*_it_’ significantly exceeds *P*_r_, *E*_FE_ becomes negative, inducing band bending that accelerates *Q*_it_’ loss toward the channel‐side (Figure 4F). MW modeling reveals that simultaneous increases in *P*_r_ and gate‐injected *Q*_it_’ effectively enhance MW characteristics (Figure S7, Supporting Information).^[^ ^46^ ^]^ However, from a retention perspective, when *Q*_it_’ exceeds *P*_r_ beyond a critical threshold, the energy band configuration becomes unstable, accelerating charge loss and degrading retention performance. Thus, achieving optimal MW and retention requires maintaining sufficiently high *P*_r_ while carefully regulating *Q*_it_’ to exceed *P*_r_ without surpassing the threshold that undermines retention stability.

**Figure 5:**
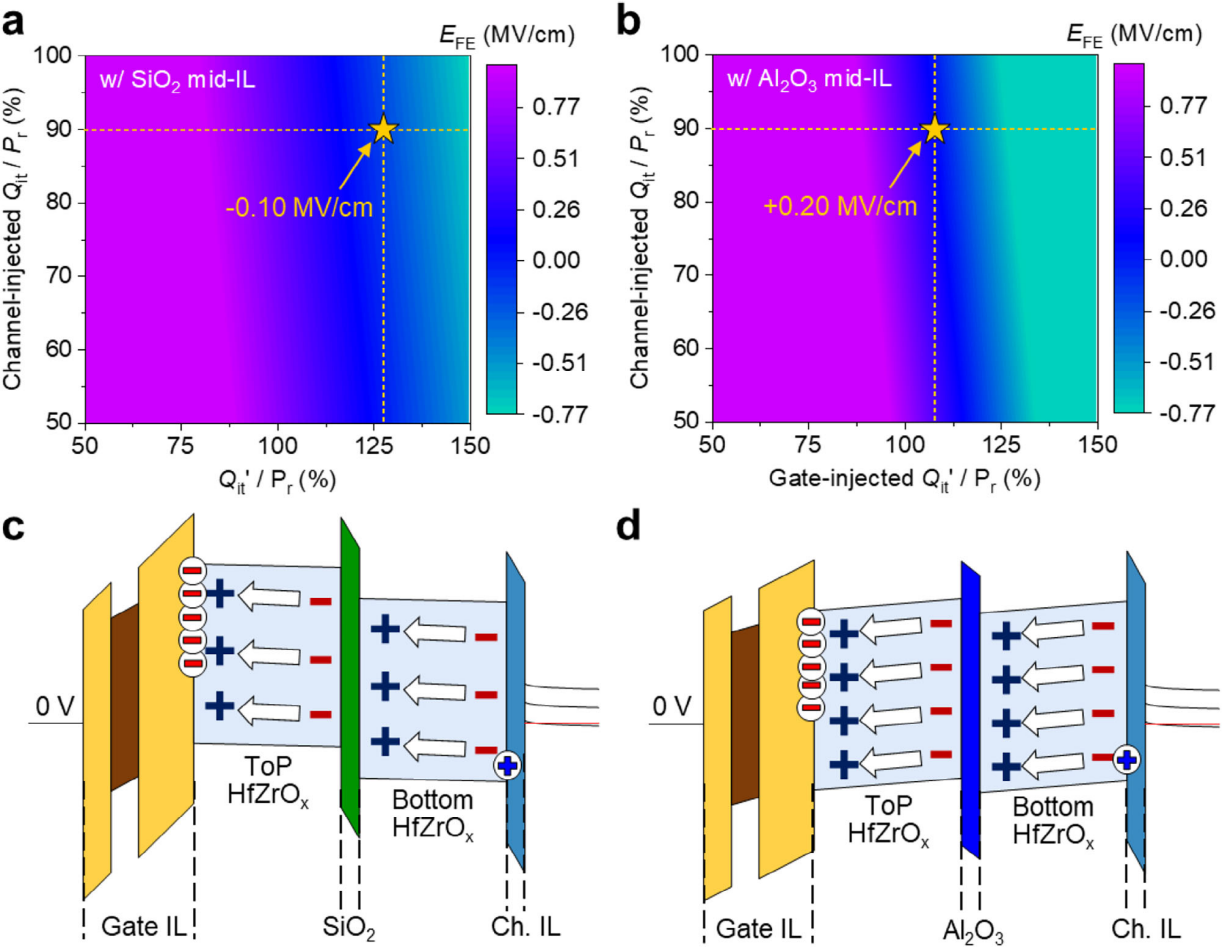
Modeling validation of retention enhancement via mid‐IL integration. Contour maps of the E_FE_ across the top HfZrO_x_ layer in the ERS state during retention for ferroelectric transistors with a) SiO_2_ and b) Al_2_O_3_ mid‐ILs. The (channel‐injected *Q*_it_)/*P*_r_ ratio is fixed at 90%, and the (gate‐injected *Q*_it_’)/*P*_r_ values required to achieve an MW of 9 V are marked by yellow stars. Simulated energy band diagrams corresponding to the retention conditions of ferroelectric transistors with c) SiO_2_ and d Al_2_O_3_ mid‐ILs, derived from experimental measurements and analytical modeling.

To assess the influence of mid‐IL integration on retention behavior, we performed an in‐depth modeling study. Initially, the (gate‐injected *Q*_it_’)/*P*_r_ ratio required to achieve a MW of 9 V, consistent with the retention conditions in Figure 4A, was determined using MW modeling (Figure S12A, Supporting Information). This extracted (gate‐injected *Q*_it_’)/*P*_r_ was then applied to the retention model (Figure S11A, Supporting Information), yielding an *E*_FE_ of −0.32 MV cm^−1^, denoted by a star symbol in Figure 4C. The negative *E*_FE_ indicates that the energy band bending within the ferroelectric layers promotes gate‐injected *Q*_it_’ loss toward the channel‐side, thereby accounting for severe retention degradation. Building on this result, we further analyzed how the incorporation of SiO_2_ and Al_2_O_3_ mid‐ILs would improve retention. Similarly, MW modeling was employed to extract the (gate‐injected *Q*_it_’)/*P*_r_ ratios necessary to achieve an initial MW of 9 V for devices with SiO_2_ and Al_2_O_3_ mid‐ILs as shown in Figure S12B,C (Supporting Information). Subsequently, these ratios were applied to the retention model (Figure S11B, Supporting Information), yielding *E*_FE_ values of −0.10 and +0.20 MV cm^−1^ for SiO_2_ and Al_2_O_3_ mid‐ILs, respectively, as marked by star symbols in **Figure** **5**A,B). For the SiO_2_ mid‐IL, its relatively low dielectric constant facilitates partial redistribution of the total electric field induced by *P*_r_, gate‐injected *Q*_it_’, and channel‐injected *Q*_it_, moderately increasing *E*_FE_. This redistribution alleviates unfavorable energy band bending, reducing charge loss and improving retention characteristics (Figure 5C). Beyond the field redistribution effect of SiO_2_, the Al_2_O_3_ mid‐IL further improves retention by enhancing the intrinsic ferroelectric properties of the HfZrO_x_ layer. In particular, the Al_2_O_3_ layer promotes higher *P*_r_, enabling a MW of 9 V without requiring excessively large gate‐injected *Q*_it_’. This favorable balance establishes an energy band alignment conducive to stable charge retention. Additionally, the increased *V*_C_ of HfZrO_x_ layers, induced by the Al_2_O_3_ mid‐IL, strengthens the resistance of polarization reversal against depolarization fields, further improving retention. The steep subloop behavior also contributes by suppressing partial polarization switching behavior triggered by *E*_FE_.^[22]^ As a result, the Al_2_O_3_ mid‐IL creates highly favorable band bending conditions for stable retention of gate‐injected *Q*_it_’. The proposed model does not fully capture transient behaviors under various thermal and electrical stress conditions. Nonetheless, the analytical modeling of key device characteristics in this study is not based on speculative assumptions but is constructed from physically formulated approaches grounded in experimentally reported spatial distributions of trapped charges.^[^ ^7^, ^8^, ^14^, ^22^, ^23^, ^24^, ^25^, ^26^, ^27^, ^38^, ^50^ ^]^ As such, the model provides essential physical insights into gate‐injection‐type ferroelectric transistors and serves as a meaningful conceptual framework for guiding future investigations.

Finally, to validate the practical applicability of gate‐injection‐type FeFETs for Fe‐NAND flash, we conducted reliability tests (Figure S13, Supporting Information). The Al_2_O_3_‐integrated ferroelectric transistor exhibits stable TLC retention exceeding 10 years at 85 °C. Moreover, compared to a conventional CTF device, the Al_2_O_3_ mid‐IL device achieves the same MW at a reduced operating voltage, demonstrating robust endurance up to 10^4^ cycles. Nevertheless, the degradation in the subthreshold slope of programmed states with increasing endurance cycles is attributed to the accumulation of channel‐injected *Q*_it_ during repeated cycles.^[^ ^55^, ^56^ ^]^ Although the demonstrated endurance of may fall short of the typical endurance requirement for TLC operation in current 3D NAND technologies (≈10^5^ cycles), the present gate‐injection‐type ferroelectric transistors can still be well suited for applications such as low‐power embedded memory and neuromorphic edge computing nodes, where frequent PGM/ERS operations are not required. On the other hand, to further enhance endurance, additional engineering of the gate metal work function may be necessary to modulate gate‐injected charge injection during PGM/ERS operations. Moreover, since the primary cause of endurance degradation in gate‐injection‐type ferroelectric transistors is attributed to the deterioration of the Ch.IL, optimizing high‐κ Ch.IL materials is also essential.^[^ ^57^, ^58^ ^]^ Finally, while conventional 3D NAND structures that rely on channel‐injected charges typically favor a gate‐all‐around (GAA) architecture, Fe‐NAND devices that operate via gate‐injected charges may benefit more from a channel‐all‐around (CAA) structure. This is because the GAA‐type structure can impose excessive electric field stress on the Ch.IL in Fe‐NANDs, potentially accelerating endurance degradation. In conclusion, the Al_2_O_3_ mid‐IL simultaneously attains wide MW, reduced *V*_PGM_, high PGM efficiency, and excellent reliability. Importantly, this study elucidates the previously unexplored retention degradation mechanism in ferroelectric transistors by combining experimental and analytical modeling, and proposes a robust gate‐stack design strategy. These insights provide new design guidelines for optimizing ferroelectric gate stacks to concurrently realize large MW and stable retention performance.

### 2.4 Gate Stack Engineered Fe‐NAND Array with Disturb‐Immunity

Thus far, device‐level characterization and gate‐stack optimization strategies have improved the performance and reliability of individual ferroelectric transistors. However, practical implementation in Fe‐NAND flash memory requires considerations beyond single‐device performance, particularly regarding disturbance characteristics. In NAND flash arrays, disturbance phenomena induce unintended *V*_th_ shifts in cells adjacent to a PGM target cell. These *V*_th_ shifts accumulate over repetitive operations, undermining data reliability, increasing power consumption due to additional error correction, and ultimately degrading system performance.^[^ ^59^, ^60^ ^]^ Despite their critical impact, disturbance mechanisms in Fe‐NAND flash have yet to be systematically investigated. In this study, we elucidate the disturbance behavior intrinsic to gate‐injection‐type FeFETs and clarify the role of mid‐IL integration through experimental and analytical modeling. Furthermore, we experimentally demonstrate Fe‐NAND array operations enabled by mid‐IL engineering.

**Figure 6:**
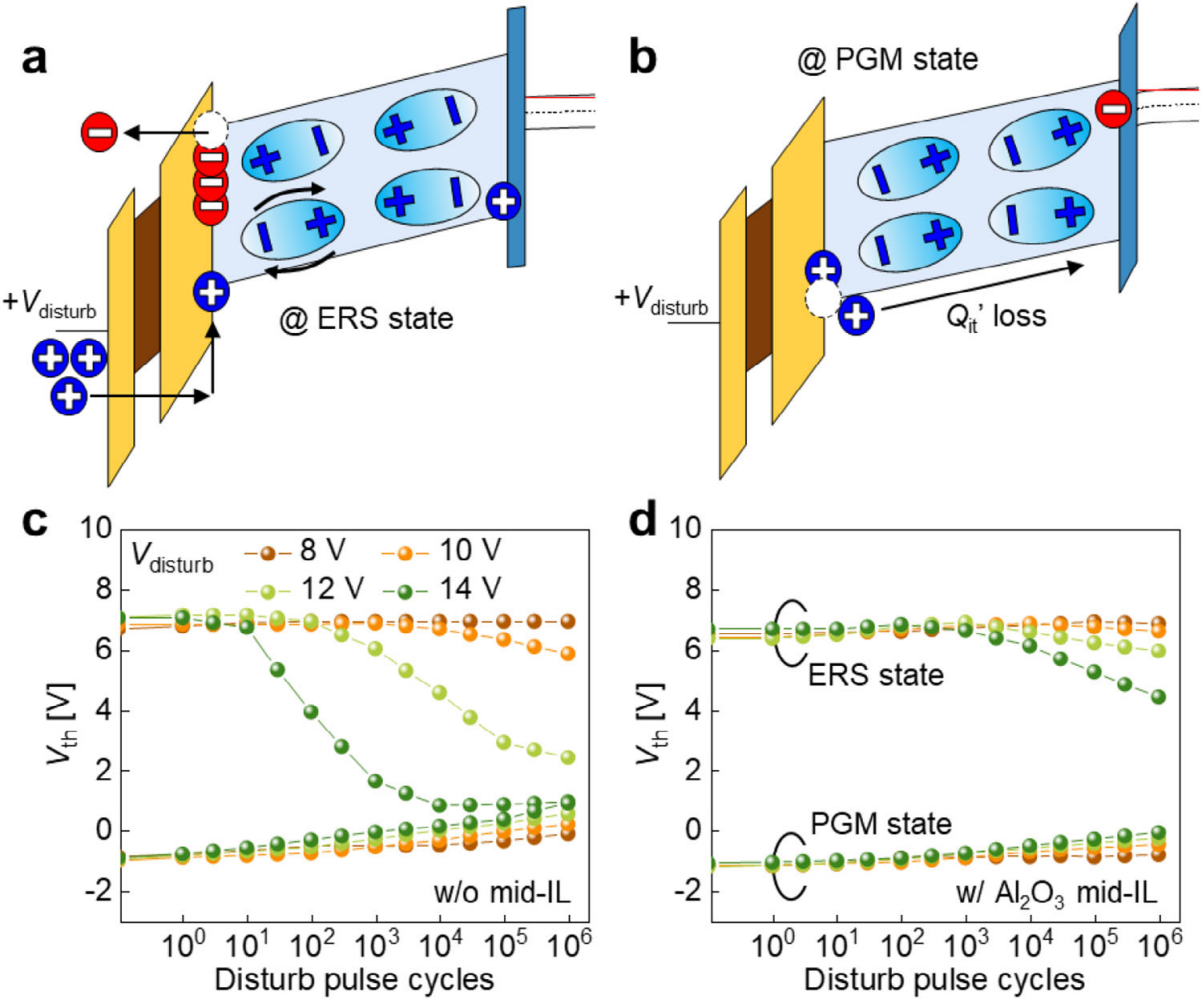
Enhanced disturbance immunity in Fe‐NAND by Al_2_O_3_ mid‐IL integration. Simulated energy band diagrams illustrating disturbance mechanisms in Fe‐NAND without a) mid‐IL under disturb voltage (*V*_disturb_) in a the ERS state and b) the PGM state. *V*_th_ variation as a function of *V*_disturb_ in ferroelectric transistors c) without and d) with Al_2_O_3_ mid‐IL, measured under pass and read conditions with a fixed pulse width of 5 µs.

To clarify the origin of disturbance‐induced *V*_th_ shifts in gate‐injection‐type ferroelectric transistors, we analyzed the energy band diagrams of MIFIS gate stacks under disturb bias (*V*_disturb_) in both ERS and PGM states as presented in **Figure** **6**A,B. It is evident that disturbance in the ERS state is significantly more pronounced than in the PGM state, irrespective of the presence of a mid‐IL. This difference is primarily governed by the type of gate‐injected *Q*_it_’ at the G.IL/HfZrO_x_ interface, as illustrated in Figure S14A,B (Supporting Information). In the ERS state, where *Q*_it_’ are electrons, the application of a positive *V*_disturb_ enables efficient hole injection from the gate into the numerous available trap sites. Furthermore, polarization reversal switching in the ERS state intensifies the disturbance. In contrast, in the PGM state, where *Q*_it_’ are holes, most interface trap sites are already occupied, making additional hole trapping under *V*_disturb_ unlikely. Additionally, polarization reversal switching does not occur under *V*_disturb_ in the PGM state, resulting in substantially improved disturbance immunity.

A more detailed analysis focusing on the interaction between charge trapping and polarization switching reveals that in the ERS state, disturbance is amplified by a self‐reinforcing feedback loop between gate‐injected *Q*_it_’ and polarization switching. Specifically, under *V*_disturb_ bias, gate‐injected *Q*_it_’ (electrons) are detrapped and escape toward the gate electrode, while holes are simultaneously injected from the gate‐side. The injected holes trigger polarization switching in the HfZrO_x_ layers, which increases band bending and facilitates further hole injection, thereby reinforcing feedback loop. In contrast, disturbance in the PGM state primarily stems from charge loss mechanisms similar to those governing retention degradation. Under *V*_disturb_, the band configuration favors gate‐injected *Q*_it_’ loss toward the channel‐side, causing *V*_th_ shifts. Therefore, effective disturbance suppression necessitates mitigating both the feedback‐driven charge injection in the ERS state and *Q*_it_’ loss in the PGM state. This can be achieved by reducing the electric fields across the gate IL (*E*_G.IL_) and *E*_FE_ under *V*_disturb_ bias.

The disturbance characteristics of MIFIS FeFETs without a mid‐IL were experimentally assessed (Figure 6C). To simulate the pass and read operations of conventional NAND flash memory, *V*_th_ shifts were measured over a range of *V*_disturb_ values from 8 to 14 V.^[^ ^5^, ^22^ ^]^ In the ERS state, the *V*_th_ shift increased significantly with rising *V*_disturb_. Conversely, the PGM state exhibited smaller shifts but higher sensitivity even at lower *V*_disturb_. Notably, integrating an Al_2_O_3_ mid‐IL markedly reduced *V*_disturb_‐induced *V*_th_ shifts in both ERS and PGM states, substantially mitigating disturbance effects (Figure 6D). This improvement is attributed to reductions in both *E*_G.IL_ and *E*_FE_ under *V*_disturb_ bias, which are enabled by the Al_2_O_3_ mid‐IL integration as shown in Figure S15A,B (Supporting Information). Modeling further confirms that both *E*_G.IL_ and *E*_FE_ are significantly lower in FeFETs with Al_2_O_3_ mid‐IL compared to the device without mid‐IL. These reductions effectively suppress both the feedback‐driven charge injection and gate‐injected *Q*_it_’ loss, thereby enhancing disturbance immunity as depicted in Figure S15C,D (Supporting Information).

**Figure 7:**
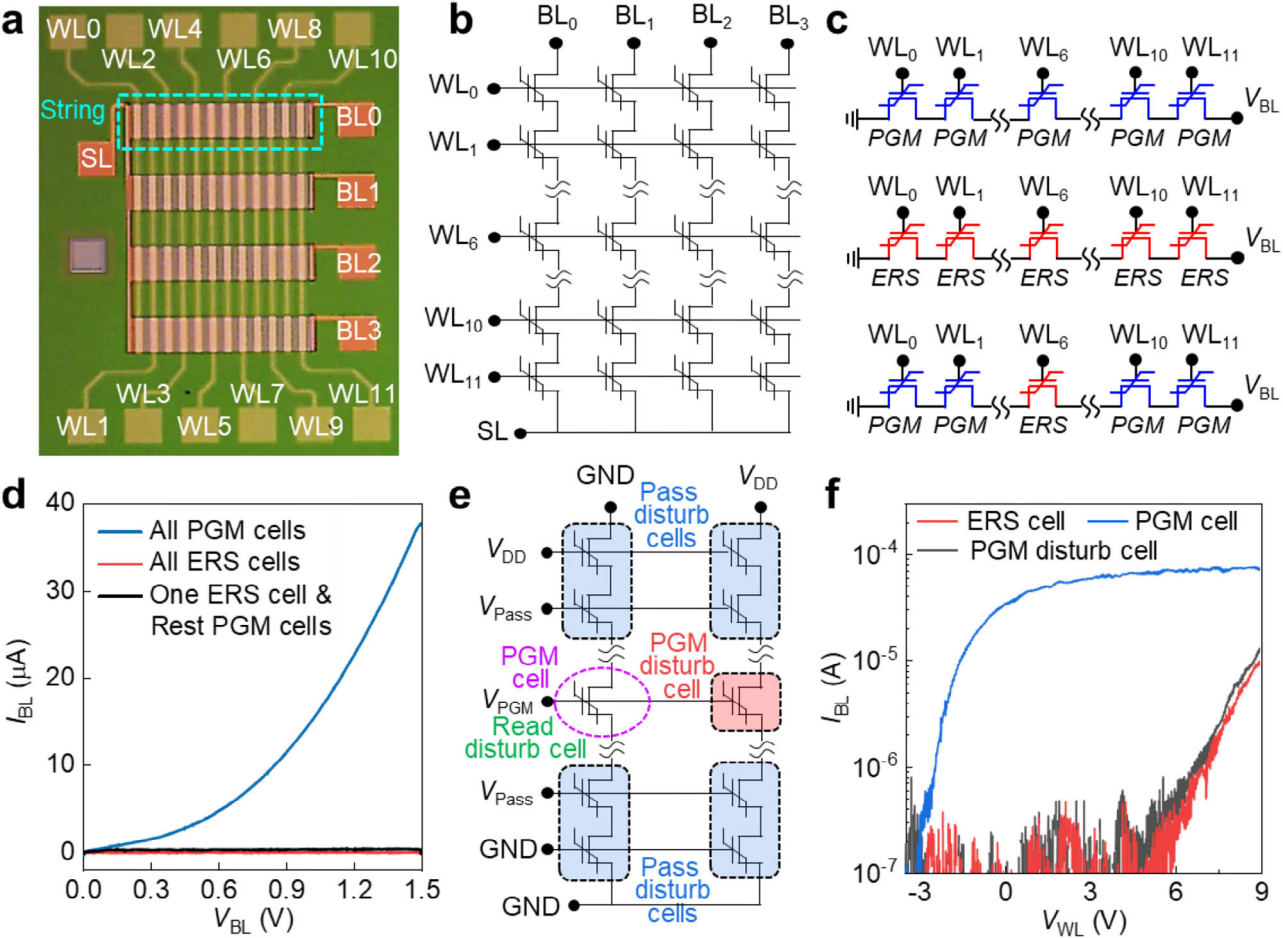
Array performance of Fe‐NAND with Al_2_O_3_ mid‐IL. a) Optical micrograph of a 12 × 4 Fe‐NAND array. b) Equivalent circuit diagram of the fabricated array. c) Verification of NAND functionality under three scenarios: all programmed cells (top), all erased cells (middle), and a single erased cell within programmed cells (bottom). d) Measured *I*_BL_‐*V*_BL_ characteristics for each scenario, confirming that the ON state is achieved only when all cells are programmed, whereas the presence of any erased cell results in the OFF state. e) Self‐boosting scheme employed to evaluate PGM disturbance characteristics. f) *I*_BL_–*V*_WL_ characteristics of PGM cell and neighboring disturb cells, demonstrating compatibility of Fe‐NAND with the self‐boosting method used in conventional NAND technologies.

Next, we verified NAND operation using the Al_2_O_3_ mid‐IL‐engineered Fe‐NAND array (**Figure** **7**A). The fabricated array consists of 12 WLs, 4 bit lines (BLs), and a common source line (SL), with its equivalent circuit shown in Figure 7B. We evaluated NAND functionality by applying three distinct scenarios to the memory strings (Figure 7C). A read current (*I*_BL_) was detected only when all cells within a string were in the PGM state (Figure 7D). In contrast, when all cells were in the ERS state, or a single ERS cell existed within a PGM string, the read operation consistently yielded an off‐state current. These results confirm the reliable NAND operation of the Al_2_O_3_ mid‐IL‐engineered Fe‐NAND array. Furthermore, during PGM operations, all cells sharing the same WL as the target cell are inherently exposed to PGM disturbance. In conventional NAND flash, this is mitigated via the global self‐boosting scheme; however, its applicability to Fe‐NAND arrays has yet to be experimentally validated. To address this, we implemented the self‐boosting scheme illustrated in Figure 7E, applying *V*_pass_ = 10 V, *V*_PGM_ = 18 V, and *V*_DD_ = 4 V. As shown in Figure 7F, the PGM target cell was selectively programmed, while adjacent cells maintained their original ERS states with negligible Δ*V*_th_. These findings demonstrate that the Al_2_O_3_ mid‐IL‐engineered Fe‐NAND array exhibits robust immunity to PGM disturbance, enabling stable PGM inhibit operation.

We established comprehensive guidelines for mid‐IL optimization by systematically analyzing its impact on MW, retention, and disturbance immunity through analytical modeling (Figure S16, Supporting Information). The modeling reveals that increasing both the *P*_r_ of HfZrO_x_ films and the dielectric constant of the mid‐IL effectively expands the MW. Conversely, retention improvement requires a higher *P*_r_ but favors a lower dielectric constant to alleviate energy band bending and suppress *Q*_it_’ loss. Similarly, disturbance immunity is enhanced by lower mid‐IL dielectric constants, which reduce both *E*_G.IL_ and *E*_FE_. Notably, the Al_2_O_3_ mid‐IL provides an optimal balance: it increases *P*_r_ while maintaining a moderate dielectric constant, simultaneously optimizing all three key metrics. This holistic gate stack strategy not only resolves the intrinsic trade‐offs among MW, retention, and disturbance immunity, but also enables reliable Fe‐NAND array operations.

Despite these promising results, the 12×4 Fe‐NAND array demonstrated in this study does not fully represent the integration scale or process complexity of high‐density 3D NAND architectures. Nevertheless, the primary objective of this work is to experimentally verify how mid‐IL engineering influences the operational characteristics of gate‐injection‐type Fe‐NAND arrays and to demonstrate that this structure serves as a promising building block capable of meeting the key technical requirements for future 3D integration. Notably, this study presents the first experimental demonstration of global self‐boosting‐based program inhibit operation and disturbance immunity in a gate‐injection‐type Fe‐NAND array, both of which are essential features for practical 3D NAND implementation.

Furthermore, the Al_2_O_3_ mid‐IL not only enhances the electrical performance of Fe‐NAND arrays but also improves their thermal stability. Previous studies have shown that HfZrO_x_ films without the Al_2_O_3_ mid‐IL undergo dielectric breakdown at thermal budgets exceeding 800 °C, whereas those incorporating Al_2_O_3_ retain robust ferroelectricity under the same conditions.^[^ ^61^ ^]^ This thermal robustness is attributed to the ability of the Al_2_O_3_ mid‐IL to physically suppress the formation of conductive breakdown paths within the HfZrOx layer during high‐temperature processing.

However, the realization of next‐generation 3D Fe‐NAND architectures (> 1000 layers) requires more than just cell device development. The thermal budget required for poly‐Si channel crystallization still poses a significant risk of degrading the ferroelectric properties of HfZrO_x_. Therefore, the development of low‐temperature processes such as metal‐induced lateral crystallization (MILC) is essential. MILC not only reduces the thermal budget to ensure compatibility with 3D integration but also helps mitigate the degradation of on‐current caused by increasing string height. In addition, achieving process reliability for 3D Fe‐NAND fabrication necessitates the development of structural supporter processes and advanced high‐aspect‐ratio contact (HARC) etching techniques. As the pitch between WLs and spacers continues to shrink, it is also critical to develop design and process technologies that can mitigate electrical interference.

In summary, this study provides the experimental validation of global self‐boosting‐based program inhibition and the contribution of Al_2_O_3_ mid‐IL to improved disturbance immunity in gate‐injection‐type Fe‐NAND arrays. These results establish essential foundational technologies that must precede full‐scale 3D NAND process development. Furthermore, the realization of scalable 3D Fe‐NAND requires not only device‐level advancements but also a comprehensive effort in process integration, structural design, and interference mitigation.

### 2.5 Mitigation of *V*_th_ Variability via Mid‐IL Integration

**Figure 8:**
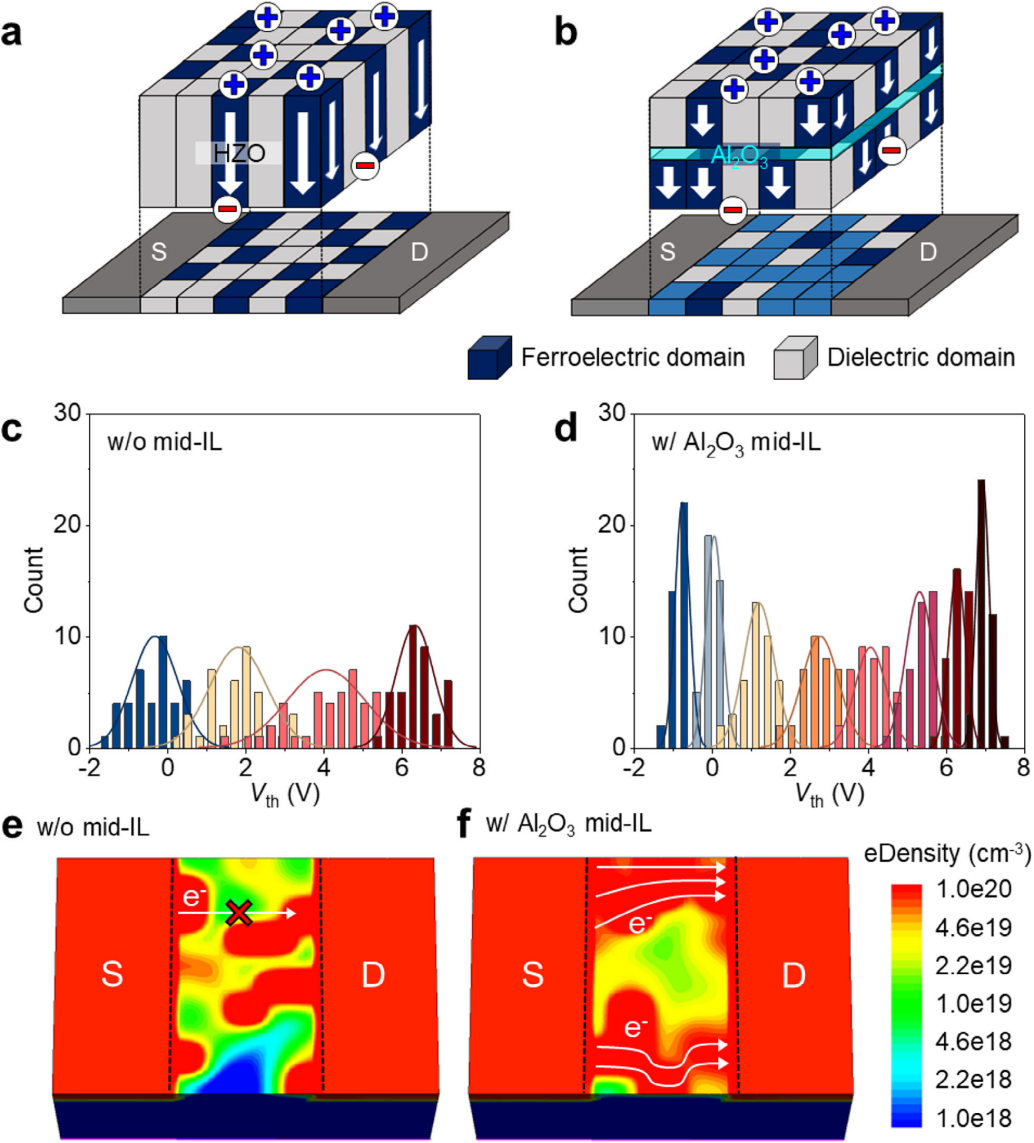
Suppression of *V*_th_ variability in Fe‐NAND via Al_2_O_3_ mid‐IL integration. Schematic illustration of ferroelectric domain configurations and resulting channel percolation effects in ferroelectric transistors a) without and b) with Al_2_O_3_ mid‐IL. The insets show corresponding *P‐*‐*V* hysteresis loops of HfZrO_x_ films used in simulations. Simulated *V*_th_ distributions for Fe‐NAND unit cell devices c) without and d) with Al_2_O_3_ mid‐IL, highlighting improved uniformity. Simulated electron density profiles in the channel during read operation at identical *V*_th_ conditions for Fe‐NAND cells e) without and f with Al_2_O_3_ mid‐IL, visualizing suppressed current percolation.

In addition to MW, retention, and disturbance immunity, *V*_th_ variability determines memory‐state distinguishability and operational reliability in Fe‐NAND flash. Compared to conventional charge‐trap‐based NAND flash, gate‐injection‐type Fe‐NAND relies on dual mechanisms, rendering it inherently susceptible to *V*_th_ variability. Specifically, polycrystalline and polymorphic HfZrO_x_ films contain randomly distributed ferroelectric domains. These domains induce local conductance non‐uniformity and promote current percolation paths.^[^ ^62^, ^63^ ^]^ The resulting percolation paths cause variations in channel conductivity, manifesting as pronounced *V*_th_ variability and limiting reliable multilevel memory operation. To mitigate this issue, we propose mid‐IL integration as a structural solution. In MIFIS FeFETs without a mid‐IL, channel conductance is strongly influenced by the stochastic distribution of ferroelectric and dielectric domains within the HfZrO_x_ layer (**Figure** **8**A). Both gate‐injected *Q*_it_’ and channel‐injected *Q*_it_ are tightly coupled to the ferroelectric domain configuration, making channel conductance highly sensitive to domain fluctuations. In contrast, the introduction of an Al_2_O_3_ mid‐IL physically decouples the top and bottom HfZrO_x_ films, allowing domains to form independently in each layer (Figure 8B). This separation averages out the impact of random domain distribution, resulting in improved channel conductance uniformity.

To quantitatively evaluate the impact of mid‐IL integration on *V*_th_ variability, we performed technology computer‐aided design (TCAD) simulations using experimentally calibrated material parameters (Figure S17, Supporting Information). Domain patterns (8 nm × 8 nm) were generated within the HfZrO_x_ layers, and ferroelectric/dielectric properties were randomly assigned based on phase ratio from GIXRD analysis (Figure 2B). Polarization switching behavior was calibrated using the Preisach model, while gate‐injected *Q*_it_’ and channel‐injected *Q*_it_ were incorporated to accurately capture the device operations. Statistical *I*_D_‐*V*_G_ simulations were iterated 40 times per state to reliably quantify *V*_th_ variability. Simulation results reveal that Fe‐NAND devices without a mid‐IL exhibit significant *V*_th_ variation (Figure 8C). Conversely, Al_2_O_3_ mid‐IL integration effectively suppresses *V*_th_ spread, yielding a tighter *V*_th_ distribution (Figure 8D). This improvement is attributed to the physical separation of the HfZrO_x_ layers by the Al_2_O_3_ mid‐IL, which enhances channel conductance uniformity. To further illustrate this effect, electron density simulations at the channel surface highlight the influence of mid‐IL integration as presented in Figure 8E,F. Without mid‐IL, localized regions of low electron density appear due to random domain‐induced percolation paths (Figure 8E). In contrast, the Al_2_O_3_ mid‐IL promotes a more uniform electron distribution (Figure 8F). The TCAD based statistical variability analysis presented in this study assumes a uniform ferroelectric domain size and does not account for stress gradients, or localized thermal effects that may arise during practical 3D NAND fabrication. However, the primary goal of this simulation is to quantitatively compare the relative impact of mid‐IL integration on V_th_ variability in HfZrO_x_ based ferroelectric transistors. The assumption of nanometer‐scale polarization domains was established based on parameters commonly used in TCAD modeling studies of ferroelectric transistors.^[^ ^64^, ^65^ ^]^ To ensure the physical relevance and comparability of the simulation, the internal ferroelectric and dielectric domain distributions in each ferroelectric transistor were defined based on the measured GIXRD analysis of HfZrO_x_ films with and without mid‐IL. These configurations were further calibrated against experimentally measured polarization switching characteristics. Additionally, key electrical parameters such as gate‐injected *Q*_it_’ and channel‐injected *Q*_it_ were incorporated into the simulation framework. Therefore, we believe this simulation provides meaningful insight into the relative trends of V_th_ variability among different MIFIS FeFET configurations. As a result, mid‐IL integration leads to a 54% improvement in the V_th_ variation of ferroelectric transistors. While this value may vary depending on actual process conditions, the results clearly demonstrate the effectiveness of mid‐IL in suppressing V_th_ variation in ferroelectric transistors.

Table S1 (Supporting Information) summarizes the performance metrics of our Al_2_O_3_ mid‐IL‐engineered Fe‐NAND flash device in comparison with state‐of‐the‐art counterparts.^[^ ^7^, ^8^, ^14^, ^21^, ^22^, ^23^, ^24^, ^25^, ^26^, ^27^, ^28^, ^38^, ^66^ ^]^ The proposed device achieves an enlarged MW at reduced operating voltages, robust retention exceeding 10 years at 85 °C, superior disturbance immunity, and a remarkable 54% reduction in *V*_th_ variability compared to conventional designs. Collectively, these results demonstrate that gate stack engineering via mid‐IL integration provides a viable approach toward highly uniform, reliable, and scalable Fe‐NAND flash memory arrays. Furthermore, beyond Fe‐NAND applications, the mid‐IL engineering strategy offers broad applicability for enhancing reliability and stability in various ferroelectric‐based memory and logic devices, including neuromorphic systems.^[^ ^67^, ^68^, ^69^, ^70^ ^]^

## 3 Discussion

We demonstrate that mid‐IL integration within the HfZrO_x_ matrix precisely modulates phase composition and domain configurations, advancing Fe‐NAND flash memory performance. The optimized Al_2_O_3_ mid‐IL confines grain growth and induces lattice strain, enhancing polarization switching both thermodynamically and kinetically. 3D phase‐field simulations confirm that mid‐IL engineering controls internal free energy and domain volume, offering a platform to tailor ferroelectric properties. Incorporating these strategies into a MIFIS gate stack strengthens polarization‐charge trapping coupling, enabling wide MW, low operating voltages, and robust TLC retention over 10 years, while ensuring scalability. Beyond single‐device performance, we address array‐level reliability concerns. Combined experiments and modeling reveal that mid‐IL insertion stabilizes energy band configurations, suppressing charge loss during retention and mitigating feedback‐driven effects under disturbance. TCAD‐based statistical simulations further show that mid‐IL integration reduces *V*_th_ variability by averaging out domain‐induced conductance fluctuations, ensuring reliable multilevel operation. While these advancements significantly improve Fe‐NAND reliability, further refinement of mid‐IL materials and gate stack design may enhance endurance. Moreover, the proposed gate stack strategy is broadly applicable to other ferroelectric‐based devices, including neuromorphic systems, where uniformity and reliability are essential. Collectively, our findings establish a scalable and reliable framework for next‐generation high‐density memory technologies.

## 4 Experimental Section

### Materials

The HfZrO_x_ layers, along with mid‐interlayers of SiO_2_, Al_2_O_3_, and TiO_2_, were deposited via plasma‐enhanced atomic layer deposition (PEALD) at 320 °C. The precursors employed were tetrakis ethylmethylamido hafnium(IV) (TEMA‐Hf) and tetrakis ethylmethylamido zirconium(IV) (TEMA‐Zr) for Hf and Zr, bisdiethylaminosilane (BDEAS) for Si, trimethylaluminum (TMA) for Al, and titanium isopropoxide (TTIP) for Ti.

### Device and Array Fabrication

For the fabrication of ferroelectric NAND flash device incorporating mid‐ILs, a 1 nm‐thick SiO_2_ channel IL was first formed via standard surfuric peroxide mixture (SPM) cleaning. To improve the quality of the channel IL, rapid thermal annealing (RTA) process was performed at 1000 °C for 30 s under a nitrogen atmosphere. Subsequently, an 8 nm‐thick HfZrO_x_ film was deposited on the SiO_2_/Si stack using atomic layer deposition (ALD) with TEMAHf, TEMAZr, and O_2_ plasma as the Hf precursor, Zr precursor, and oxygen source, respectively. The ALD cycle ratio of HfO_2_ to ZrO_2_ was maintained at 1:1 to form the desired Hf_0.5_Zr_0.5_O_2_ composition. Following the initial HfZrO_x_ deposition, mid‐IL layers comprising SiO_2_, Al_2_O_3_, and TiO_2_ were deposited using BDEAS, TMA, and TTIP as the Si, Al, and Ti precursors, respectively. Additional HfZrO_x_ layers were then deposited on top of the mid‐ILs by using the same ALD process. For the gate IL formation, a 3 nm‐thick SiO_2_ layer was deposited, followed by a 1.5 nm‐thick SiN layer using BDEAS and NH_3_ plasma as the Si precursor and nitrogen source, respectively. An additional 1.5 nm‐thick SiO_2_ layer was deposited to complete the SiO_2_/SiN/SiO_2_ gate IL structure. Finally, the gate metal electrodes for unit cell devices and the interconnection for the Fe‐NAND array were completed by TiN/Al metallization using DC sputtering. The crystallization annealing was carried out by RTA at 600 °C for 10 s in a nitrogen atmosphere to induce the ferroelectric phase in the HfZrO_x_ films.

### Characterization

The polarization response of the fabricated ferroelectric capacitors was characterized using a semiconductor parameter analyzer (4200A‐SCS, Keithley). For the ferroelectric transistors and Fe‐NAND arrays, pulse‐based measurements were conducted to emulate the operation conditions of commercial NAND flash memory. A probe station, pulse generator (81110A, Agilent), and device current waveform analyzer (CX‐3324A, Keysight) were utilized to perform these pulse measurements. Retention and gate‐biased retention tests were carried out at 85 °C in an ambient atmosphere, whereas all other electrical characterizations were conducted at room temperature under ambient conditions. Sentaurus TCAD (Synopsys Inc.) software was used for the device simulation.

## Conflict of Interest

The authors declare no conflict of interest.

## Author Contributions

G.K. and S.J. conceived the idea and designed the experiments. S.L. and Y.J. performed the device simulation. G.K and H.C. fabricated the devices and performed the electrical measurements and data analysis. G.K. wrote the manuscript. J.A. and S.J. supervised the research. All authors discussed the results and commented on the manuscript.

## Supporting information



Supporting Information

## Data Availability

The data that support the findings of this study are available on request from the corresponding author. The data are not publicly available due to privacy or ethical restrictions.
