# Supplementary material for: Middle Interlayer Engineered Ferroelectric NAND Flash Overcoming Reliability and Stability Bottlenecks for Next‐Generation High‐Density Storage Systems
Source: Adv Sci (Weinh). 2025 Aug 23;12(40):e10155. doi: 10.1002/advs.202510155 (PMC12561253; doi:10.1002/advs.202510155)

Supplementary Information

**Middle interlayer engineered ferroelectric NAND flash overcoming reliability and stability bottlenecks for next-generation high-density storage systems**

Giuk Kim^1^, Sangho Lee^1^, Hyojun Choi^1^, Yangjin Jung^1^, Sanghyun Park^2^, Kwangyou Seo^2^, Kwangsoo Kim^2^, Wanki Kim^2^, Daewon Ha^2^, Mincheol Shin^1^, Jinho Ahn^3^*****, and Sanghun Jeon^1^*****

^1^Department of Electrical engineering, Korea Advanced Institute of Science and Technology (KAIST), Daejeon, 34141, Korea.

^2^Semiconductor R&D Center, Samsung Electronics, Hwaseong-si, 18367, Korea.

^3^Department of Material Science Engineering, Hanyang University, Seoul, 04763, Korea.

*E-mail: jhahn@hanyang.ac.kr and jeonsh@kaist.ac.kr

**Supplementary Text**

1. Analytical MW equation of gate-injection type ferroelectric transistors depending on different middle interlayer (mid-IL) properties

Analytical equation for memory window (MW) of gate-injection-type ferroelectric transistors, based on previous studies, was derived as a simple function of polarization, gate-injected *Q*_it_’, and channel-injected *Q*_it_ (*8, 46*). Here, gate-injected *Q*_it_’ is defined as the interface-trapped charge formed at the gate interlayer (gate IL)/HfZrO_x_ interface by carriers injected from the gate-side, while channel-injected *Q*_it_ refers to the interface-trapped charge accumulated at the channel interlayer (channel IL)/HfZrO_x_ interface via carrier injection from the channel-side. Supplementary Fig. 7A illustrates the charge distribution in the metal-gate IL-ferroelectrics-channel IL-semiconductor (MIFIS) gate stack under flat-band voltage (*V*_FB_) bias at the program (PGM) state. Under this condition, the charge distribution is modeled using delta functions, as detailed below:

$$\rho\left( x \right)\left\{ \begin{aligned} (Q_{it}^{'}-P_{r})\cdot\delta(x-t_{G.IL}) \\ (P_{r}-Q_{it})\cdot\delta(x-(t_{G.IL}+t_{FE}) \end{aligned} \right.$$

where *P*_r_ is the remnant polarization, *t*_G.IL_ and *t*_FE_ denote the thickness of the gate IL and ferroelectric layer, respectively. Poisson’s equation is employed to quantify the band bending in the silicon channel induced by the gate stack charges in the PGM or erase (ERS) state. By applying the *V*_FB_ to the gate, the channel is flattened, allowing the voltage drop across the channel, solely attributed to the gate stack charges, to be determined as following equation:

$${\Delta V}_{FB,PGM}=-\int_{0}^{t} Edx=\int_{0}^{t_{G.IL}+t_{FE}+t_{Ch.IL}} -\frac{1}{\varepsilon\left( x \right)}[x\left( Q_{it}^{'}-P_{r} \right)\cdot\delta\left( x-t_{G.IL} \right)$$

$$+x\left( P_{r}-Q_{it} \right)\cdot\delta\left( x-t_{G.IL}-t_{FE} \right)]dx$$

where $\varepsilon$_G.IL_ and $\varepsilon$_FE_ denote the dielectric constant of the gate IL and ferroelectric layer. The MW is defined as the difference between *V*_th,PGM_ and *V*_th,ERS_, which can be approximated by the difference between *V*_FB,PGM_ and *V*_FB,ERS_. Accordingly, the analytical expression for MW is derived as follows:

$$MW\approx{\Delta V}_{FB,PGM}-{\Delta V}_{FB,ERS}=\frac{\Delta P_{r}}{C_{FE}}+\frac{{\Delta Q}_{it}^{'}}{C_{G.IL}}-\frac{{\Delta Q}_{it}}{\left( \frac{1}{C_{FE}}+\frac{1}{C_{G.IL}} \right)^{-1}}$$

where *C*_FE_ and *C*_G.IL_ denote the capacitance of the ferroelectric layer and gate IL. A higher *P*_r_ and larger gate-injected *Q*_it_’, combined with a lower channel-injected *Q*_it_, lead to an enhanced MW characteristic in gate-injection–type ferroelectric transistors.

In contrast, Supplementary Fig. 7B depicts the charge distribution in the MIFIS gate stack with mid-ILs possessing lower dielectric constants compared to the HfZrO_x_ film (e.g., SiO_2_, Al_2_O_3_) under *V*_FB_ bias in the PGM state. Under this condition, the charge distribution within the gate stack is represented as below:

$$\rho\left( x \right)\left\{ \begin{aligned} (Q_{it}^{'}-P_{r})\cdot\delta(x-t_{G.IL}) \\ P_{r}\cdot\delta(x-(t_{G.IL}+t_{FE1}) \\ {-P}_{r}\cdot\delta\left( (x-\left( t_{G.IL}+t_{FE1}+t_{mid-IL} \right) \right) \\ (P_{r}-Q_{it})\cdot\delta\left( x-\left( t_{G.IL}+t_{FE1}+t_{mid-IL}+t_{FE2} \right) \right) \end{aligned} \right.$$

where *t*_FE1_, *t*_FE2_, and *t*_mid-IL_ represent the thickness of the top and bottom ferroelectric layers, and mid-IL, respectively. Following the same procedure, the MW equation for ferroelectric transistors with SiO_2_ and Al_2_O_3_ mid-ILs is derived, as follows:

$$MW\approx\frac{\Delta P_{r}}{\left( \frac{1}{C_{FE1}}+\frac{1}{C_{FE2}} \right)^{-1}}+\frac{{\Delta Q}_{it}^{'}}{C_{G.IL}}-\frac{{\Delta Q}_{it}}{\left( \frac{1}{C_{G.IL}}+\frac{1}{C_{FE1}}+\frac{1}{C_{mid-IL}}+\frac{1}{C_{FE2}} \right)^{-1}}$$

where *C*_FE1_, *C*_FE2_, and *C*_mid-IL_ denote the capacitance of the top ferroelectric layer, bottom ferroelectric layer and mid-IL.

Lastly, Supplementary Fig. 7C presents the charge distribution in the MIFIS gate stack containing mid-ILs with higher dielectric constants than the HfZrO_x_ film, such as TiO_2_, under *V*_FB_ bias at the PGM state. In this case, the charge distribution is modeled using delta functions, as detailed below:

$$\rho\left( x \right)\left\{ \begin{aligned} (Q_{it}^{'}-P_{r})\cdot\delta(x-t_{G.IL}) \\ P_{r}\cdot\delta(x-(t_{G.IL}+t_{FE1}) \\ -Q_{well}\cdot\delta(x-(t_{G.IL}+t_{FE1}+\frac{t_{mid-IL}}{2}) \\ {-P}_{r}\cdot\delta\left( (x-\left( t_{G.IL}+t_{FE1}+t_{mid-IL} \right) \right) \\ (P_{r}-Q_{it})\cdot\delta\left( x-\left( t_{G.IL}+t_{FE1}+t_{mid-IL}+t_{FE2} \right) \right) \end{aligned} \right.$$

where *Q*_well_ denotes the trapped charge within the potential well formed between the top and bottom ferroelectric layers by the high-κ mid-IL. The corresponding analytical equation for the MW of ferroelectric transistors with TiO_2_ mid-IL is obtained following the same procedure, as derived below:

$$MW\approx\frac{\Delta P_{r}}{\left( \frac{1}{C_{FE1}}+\frac{1}{C_{FE2}} \right)^{-1}}+\frac{{\Delta Q}_{it}^{'}}{C_{G.IL}}-\frac{{\Delta Q}_{it}}{\left( \frac{1}{C_{G.IL}}+\frac{1}{C_{FE1}}+\frac{1}{C_{mid-IL}}+\frac{1}{C_{FE2}} \right)^{-1}}-\frac{{\Delta Q}_{well}}{\left( \frac{1}{C_{G.IL}}+\frac{1}{C_{FE1}}+\frac{1}{{2C}_{mid-IL}} \right)^{-1}}$$

2. Electric field across the ferroelectric layer (*E*_FE_) induced by the charges within the MIFIS gate stack under retention conditions, depending on the selection of mid-IL

Retention degradation is primarily attributed to the loss of gate-injected *Q*_it_’, which subsequently migrates toward the channel-side. Therefore, it is essential to understand the energy band diagram of the MIFIS gate stack. In this regard, the electric field across the ferroelectric layer (*E*_FE_) serves as a key parameter, as it reflects how band bending within the ferroelectric layer is governed by *P*_r_, gate-injected *Q*_it_’, and channel-injected *Q*_it_ under retention conditions. To derive a more intuitive and simple equation for quantifying *E*_FE_, we employ a charge-sheet model to evaluate *E*_FE_ as an indicator of retention characteristics in ferroelectric transistors. Since retention degradation in gate-injection-type ferroelectric transistors predominantly occurs in the ERS state, our retention modeling study focuses on quantifying *E*_FE_ specifically in the ERS state. Supplementary Fig. 11(A) illustrates the charge distribution in the MIFIS gate stack without a mid-IL under retention conditions (*V*_G_ = 0 V) in the ERS state. In this case, the electric field distribution in the MFMIS gate stack can be expressed based on the conservation of electric displacement, as described by the following equation:

$$\left\{ \begin{aligned} D=P_{r}-Q_{it}^{'}+\varepsilon_{G.IL}\cdot E_{G.IL}=\varepsilon_{FE}\cdot E_{FE} \\ D={-P}_{r}+Q_{it}+\varepsilon_{FE}\cdot E_{FE}=\varepsilon_{Ch.IL}\cdot E_{Ch.IL} \end{aligned} \right.$$

where *D* represents the electric displacement, $\varepsilon$_Ch.IL_ denotes the dielectric constant of the channel IL, *E*_G.IL_ and *E*_Ch.IL_ refer to the electric field across the gate IL and channel IL, respectively. Furthermore, under the retention condition with *V*_G_ of 0 V, the voltage induced by the charges within the gate stack is distributed across each component of the gate stack (8). This voltage distribution can be approximated by the following relation:

$$t_{G.IL}\cdot E_{G.IL}+t_{FE}\cdot E_{FE}+t_{Ch.IL}\cdot E_{Ch.IL}\approx0$$

where *t*_Ch.IL_ denotes the thickness of the channel IL. Finally, by substituting​ *E*_G.IL_ and *E*_Ch.IL_ obtained from the conservation of electric displacement relation into the voltage distribution equation, the *E*_FE_ under retention conditions can be expressed as a function of the *P*_r_, gate-injected *Q*_it_’, and channel-injected *Q*_it_, as follows:

$$E_{FE}\approx\frac{\left( P_{r}-Q_{it} \right)}{\varepsilon_{FE}\left( \frac{1}{C_{total}}\cdot\left( \frac{1}{C_{G.IL}}+\frac{1}{C_{Ch.IL}} \right) \right)^{-1}}-\frac{\left( Q_{it}^{'}-Q_{it} \right)}{\varepsilon_{FE}\left( \frac{1}{C_{total}}\cdot\frac{1}{C_{G.IL}} \right)^{-1}}$$

where *C*_total_ and *C*_Ch.IL_ refer to the total capacitance of gate stack and channel IL, respectively.

On the other hand, Supplementary Fig. 11(B) illustrates the charge distribution under retention conditions in the ERS state of the MIFIS gate stack, where a mid-IL is inserted between the top and bottom ferroelectric layers. Similar to the previous case, the electric field distribution within the gate stack can be described using the conservation of electric displacement, as expressed by the following equation:

$$\left\{ \begin{aligned} D=P_{r}-Q_{it}^{'}+\varepsilon_{G.IL}\cdot E_{G.IL}=\varepsilon_{FE1}\cdot E_{FE1} \\ D={-P}_{r}+\varepsilon_{FE1}\cdot E_{FE1}=\varepsilon_{mid-IL}\cdot E_{mid-IL} \\ D=\varepsilon_{mid-IL}\cdot E_{mid-IL}=P_{r}+\varepsilon_{FE2}\cdot E_{FE2} \\ D={Q_{it}-P}_{r}+\varepsilon_{FE2}\cdot E_{FE2}=\varepsilon_{Ch.IL}\cdot E_{Ch.IL} \end{aligned} \right.$$

where $\varepsilon$_FE1,_ $\varepsilon$_FE2,_ and $\varepsilon$_mid-IL_ represent the dielectric constant of the top ferroelectric layer, bottom ferroelectric layer, and mid-IL, *E*_FE1_, *E*_FE2_, and *E*_mid-IL_ denote the electric field across the top ferroelectric layer, bottom ferroelectric layers and mid-IL, respectively. Since top and bottom ferroelectric layers share the same composition ratio (Hf:Zr of 1:1) and fabrication conditions, they are assumed to be identical in this analysis. Additionally, in the retention, the voltage generated by the gate stack charges is shared across each layer of the gate stack. The voltage distribution follows the relation given below:

$$t_{G.IL}\cdot E_{G.IL}+t_{FE1}\cdot E_{FE1}+t_{mid-IL}\cdot E_{mid-IL}+t_{FE2}\cdot E_{FE2}+t_{Ch.IL}\cdot E_{Ch.IL}\approx0$$

Finally, substituting *E*_G.IL_, *E*_mid-IL_, and *E*_Ch.IL_, derived from the electric displacement continuity condition, into the voltage distribution equation allows the *E*_FE_ under retention conditions to be expressed as follows:

$$E_{FE}\approx\frac{\left( P_{r}-Q_{it} \right)}{\varepsilon_{FE1}\left( \frac{1}{C_{total}}\cdot\left( \frac{1}{C_{G.IL}}+\frac{1}{C_{Ch.IL}} \right) \right)^{-1}}-\frac{\left( Q_{it}^{'}-Q_{it} \right)}{\varepsilon_{FE1}\left( \frac{1}{C_{total}}\cdot\frac{1}{C_{G.IL}} \right)^{-1}}+\frac{P_{r}}{\varepsilon_{FE1}\left( \frac{1}{C_{total}}\cdot\frac{1}{C_{mid-IL}} \right)^{-1}}$$

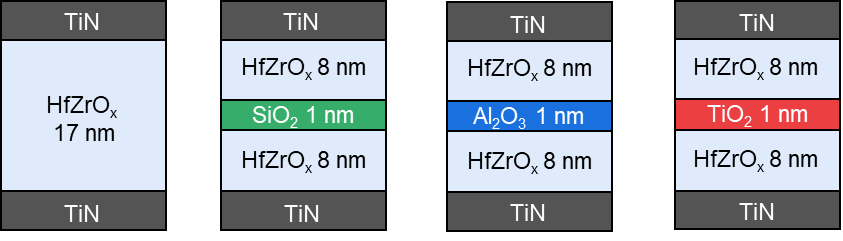


Supplementary Figure 1. Structures of HfZrOx capacitors with and without mid-IL. Schematic illustration of the TiN/HfZrO_x_/TiN capacitor structures used to investigate polarization dynamics, highlighting the configurations without mid-IL and with SiO_2_, Al_2_O_3_, or TiO_2_ mid-ILs inserted between two HfZrO_x_ matrixes.


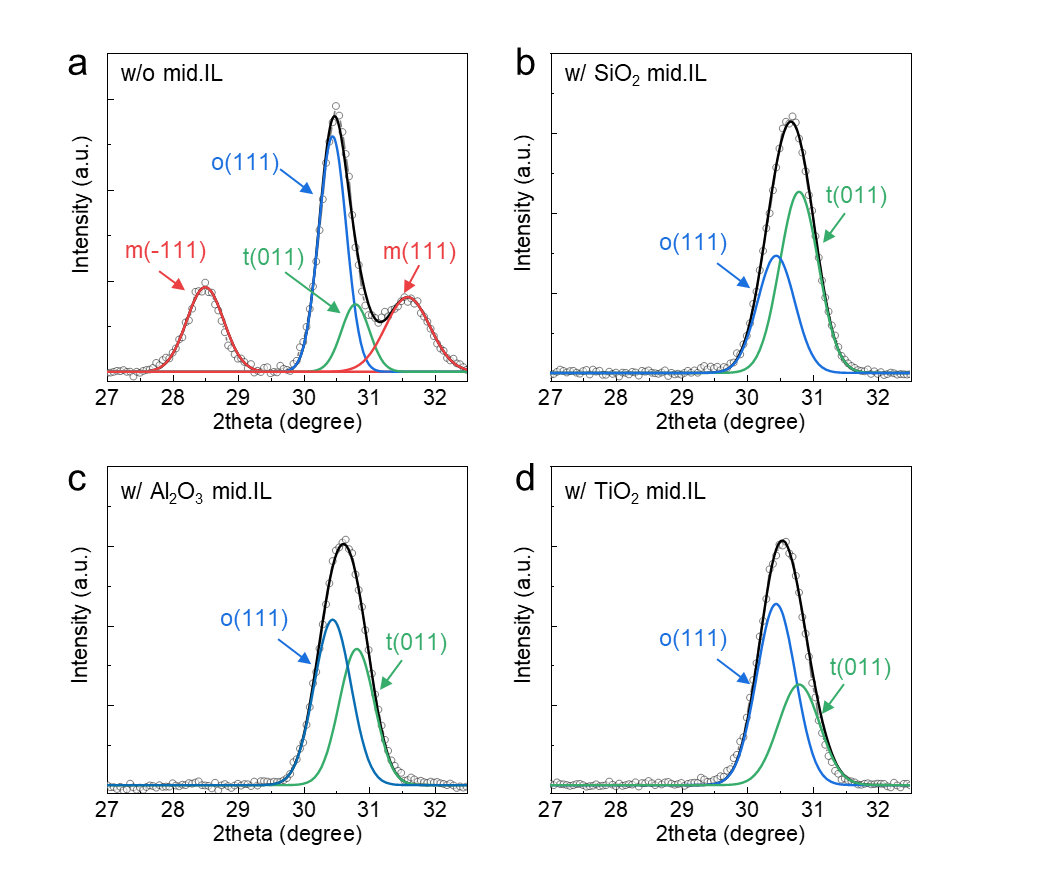


Supplementary Figure 2. Phase analysis of HfZrO_x_ films with varying mid-IL. GIXRD spectra and corresponding deconvoluted peaks of HfZrO_x_ films (a) without a mid-IL and with (b) SiO_2_, (c) Al_2_O_3_, and (d) TiO_2_ mid-ILs. Black symbols denote the raw grazing incidence X-ray diffraction (GIXRD) data. The well-defined diffraction peaks of the monoclinic phase (m-phase, m(-111) and m(111)) are clearly distinguished from the overlapping peaks of the orthorhombic (o-phase, o(111)) and tetragonal (t-phase, t(011)) phases. The o(111) and t(011) peaks are assigned to 30.4° and 30.8°, respectively, following previous reports (*70*). Notably, the introduction of a mid-IL suppresses the m-phase and promotes o-phase stabilization, with the effect most pronounced in the order of SiO_2_, Al_2_O_3_, and TiO_2_.


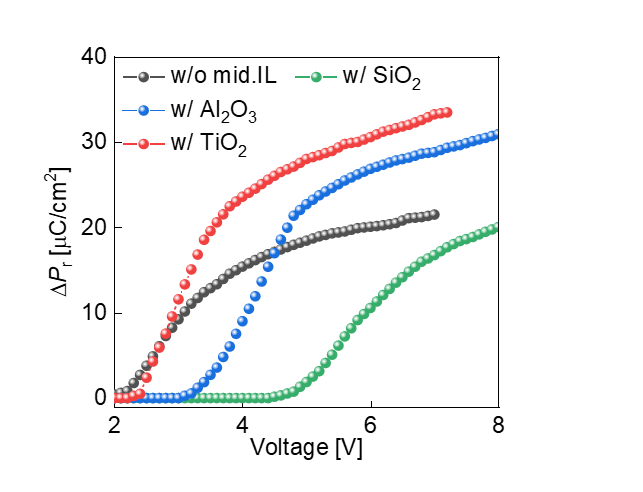


Supplementary Figure 3. Polarization switching behavior of HfZrO_x_ with and without mid-ILs. Variation of *P*_r_ ($\Delta$*P*_r_) as a function of voltage for HfZrO_x_ films incorporating SiO_2_, Al_2_O_3_, and TiO_2_ mid-ILs, compared to the control device without a mid-IL. The presence and type of mid-IL significantly modulate *P*_r_, partial polarization switching behavior, and coercive voltage (*V*_C_).


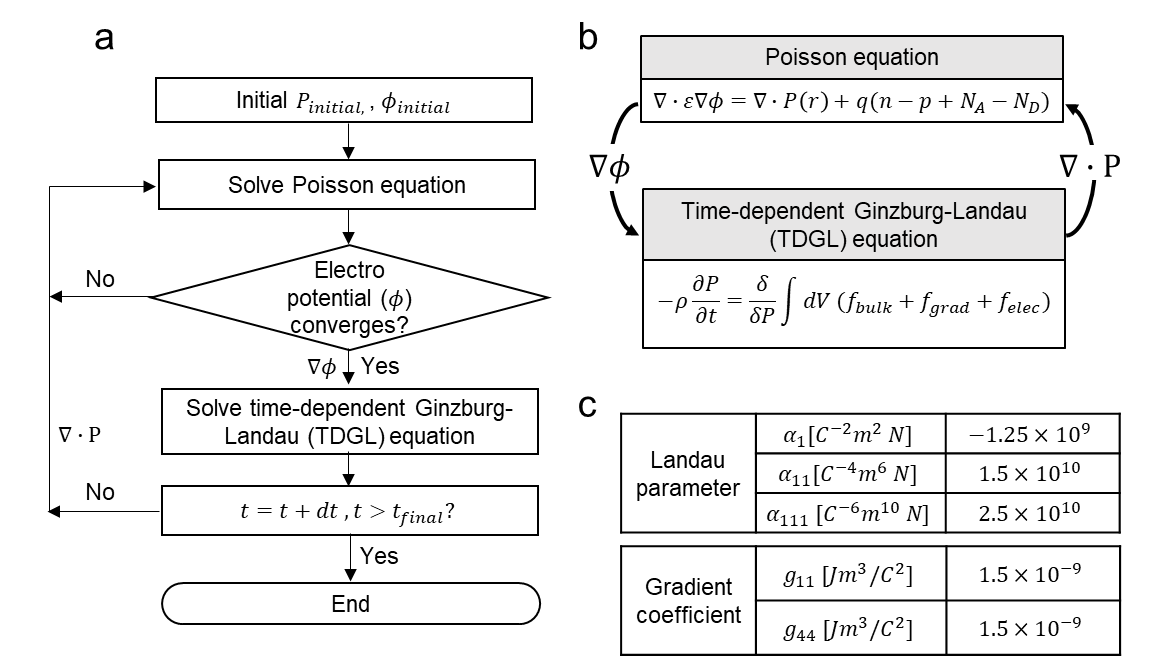


Supplementary Figure 4. Framework of three-dimensional (3D) phase-field simulations. (a) Schematic illustration of the modeling framework and algorithmic flow used for 3D phase-field simulations. *P*_initial_ denotes initial polarization and $\phi$_initial_ represents the initial electro potential. (b) Coupled iteration process solving Poisson equation and the time-dependent Ginzburg-Landau (TDGL) equation to evaluate the free energy profile of HfZrO_x_ films with and without mid-ILs. (c) Material parameters and boundary conditions used in the simulation were referenced and adapted from previously reported studies on hafnia-based ferroelectric systems (33).


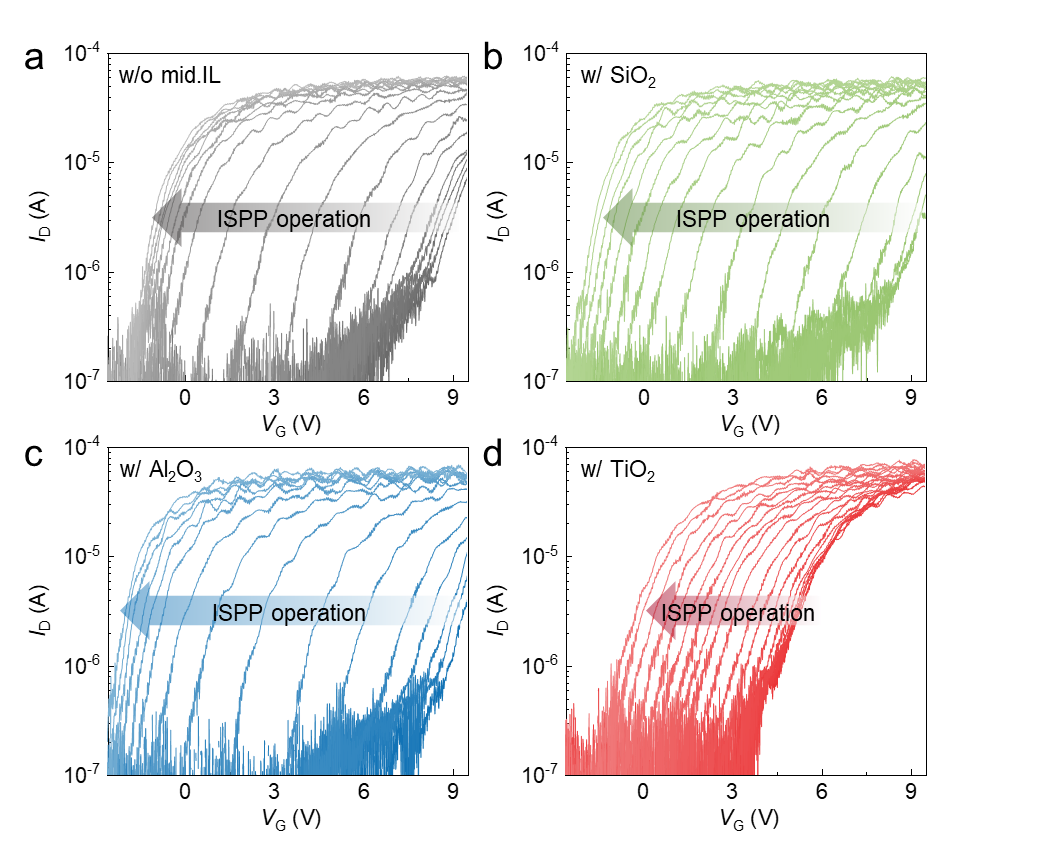


Supplementary Figure 5. MW characterization via incremental step pulse programming (ISPP) operation of ferroelectric transistors with and without mid-IL. Measured pulse *I*_D_-*V*_G_ curves during ISPP operation for ferroelectric transistors (a) without mid-IL, (b) with SiO_2_ mid-IL, (c) with Al_2_O_3_ mid-IL, and (d) with TiO_2_ mid-IL. Each ISPP pulse has a width of 10 μs, with its amplitude incremented stepwise by 0.5 V.


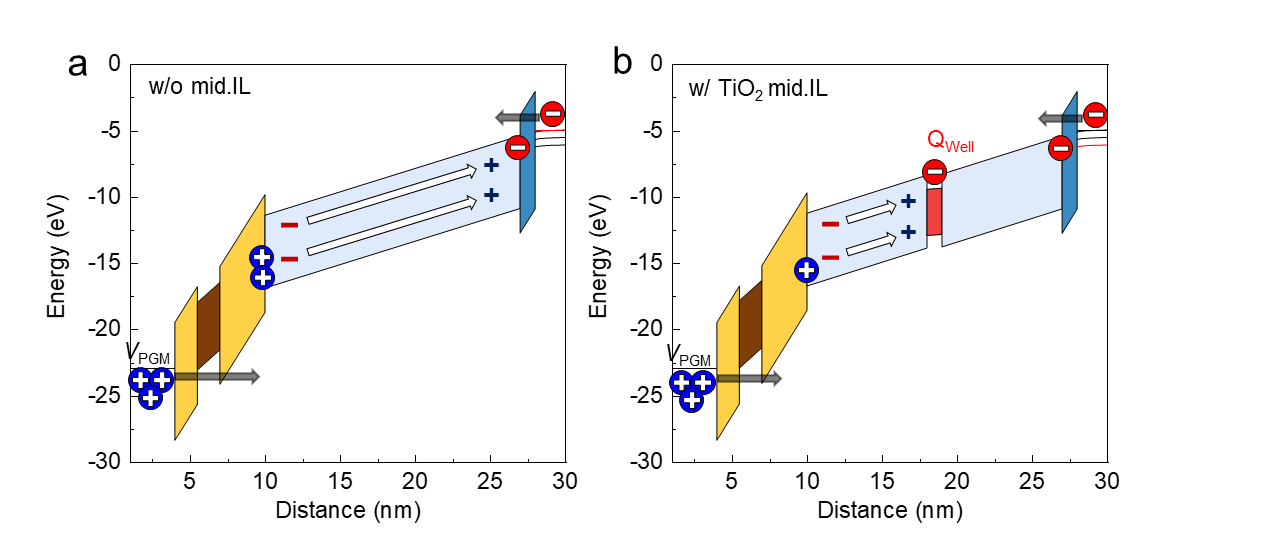


Supplementary Figure 6. Energy band comparison of MIFIS gate stack with and without TiO_2_ mid-IL under PGM operation. Simulated energy band diagrams of ferroelectric transistors (a) without and (b) with TiO_2_ mid-IL under program operation. While the TiO_2_ mid-IL effectively stabilizes the o-phase in HfZrO_x_, enhancing *P*_r_ and sub-loop behavior, its high-κ properties induce a potential well between top and bottom HfZrO_x_ layers. This well promotes the generation of additional trapped charges (*Q*_well_), which disrupt polarization switching and ultimately narrow the MW.


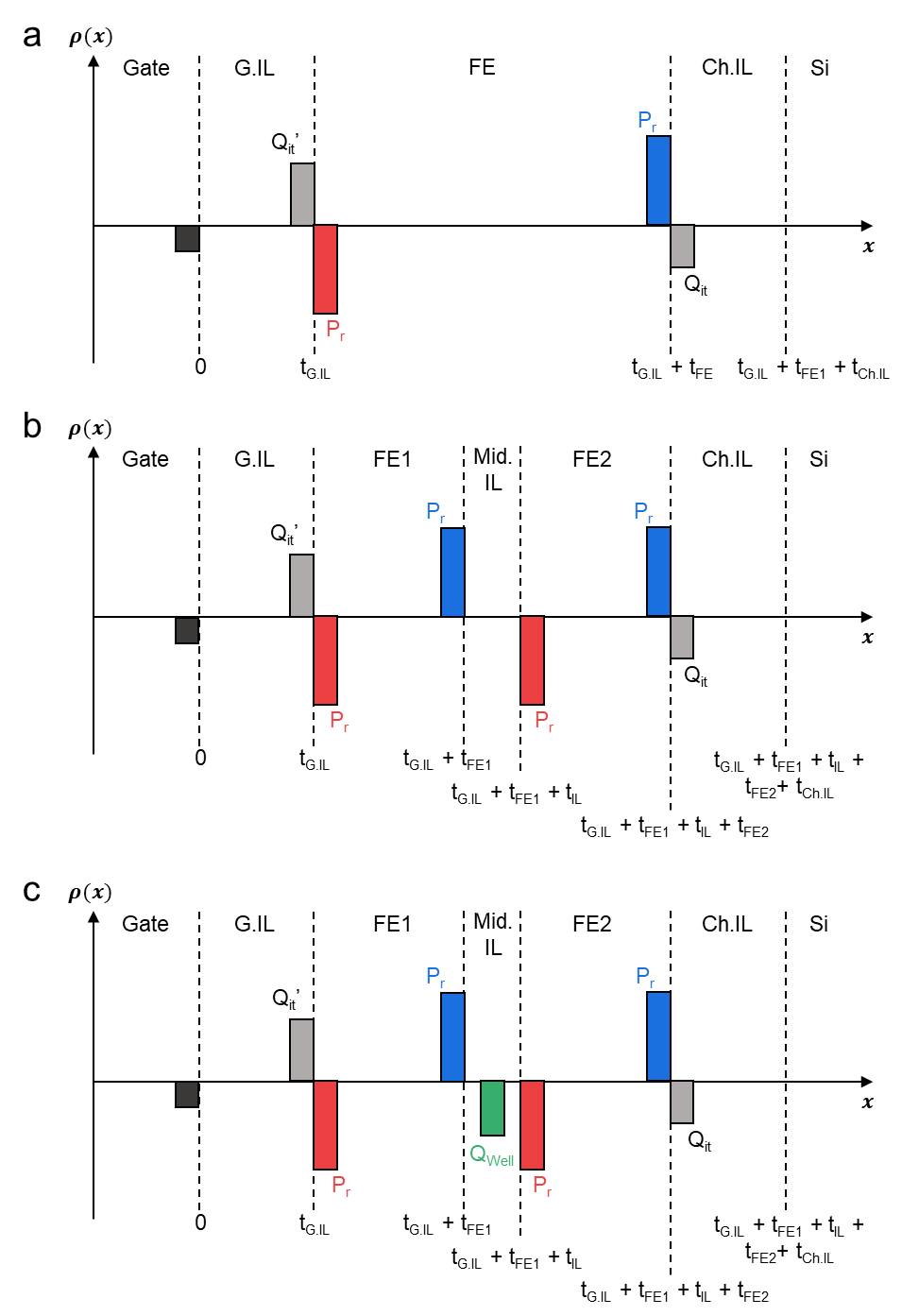


Supplementary Figure 7. Charge-sheet modeling to analyze the impact of mid-IL on MW. Schematic illustration of charge distribution in MIFIS gate stacks under flat-band voltage bias at the PGM state: (a) without a mid-IL, (b) with a mid-IL having a lower dielectric constant than HfZrO_x_ film (e.g., SiO_2_, Al_2_O_3_), and (c) with a mid-IL possessing a higher dielectric constant than HfZrO_x_ (e.g., TiO_2_). Refer to Supplementary Text 1 for additional details.


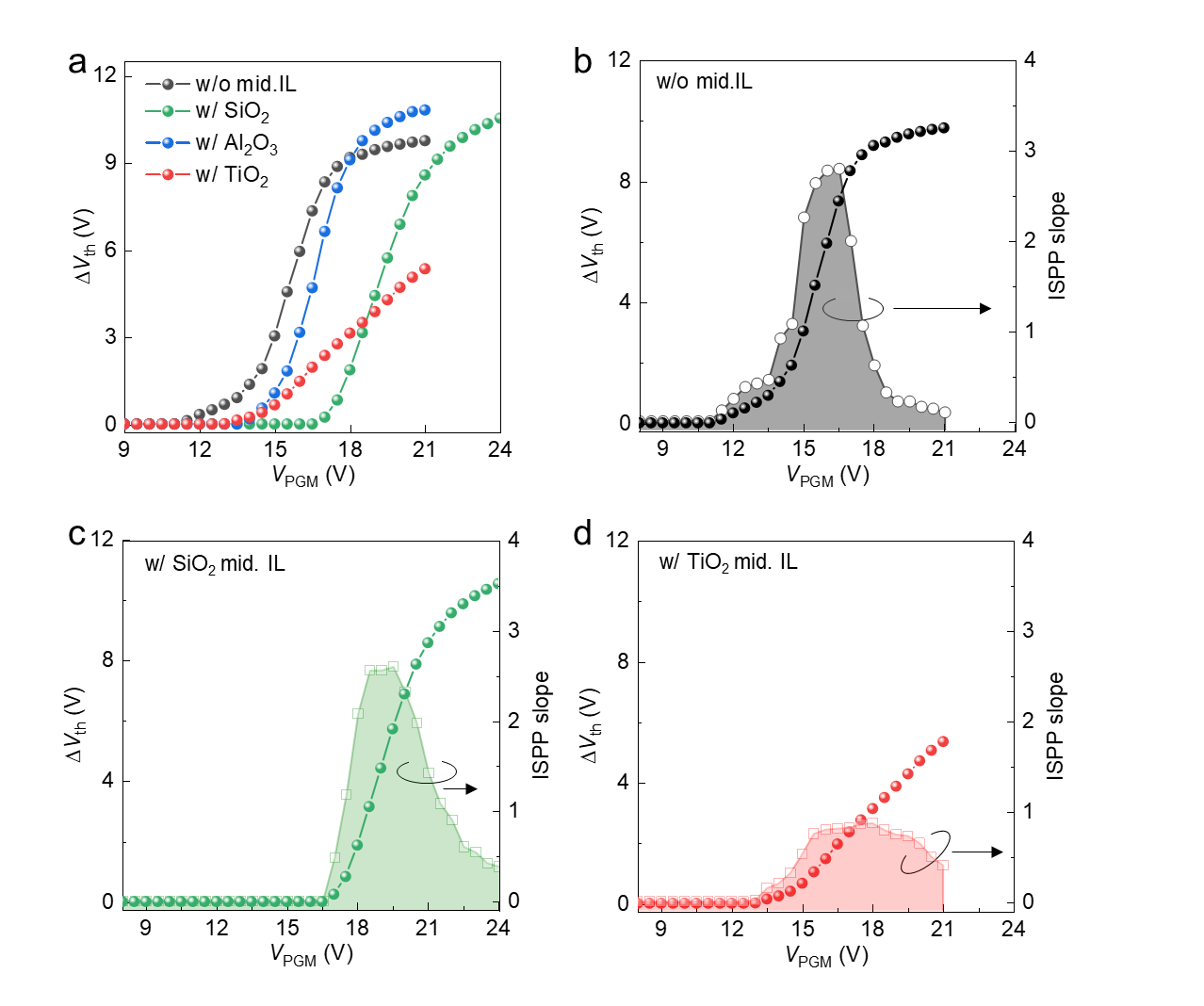


Supplementary Figure 8. ISPP performance analysis of ferroelectric transistors with and without mid-ILs. (a) Comparison of $\Delta$*V*_th_ evolution as a function of program voltage (*V*_PGM_) for ferroelectric transistors without a mid-IL and with SiO_2_, Al_2_O_3_, and TiO_2_ mid-ILs. Extracted $\Delta$*V*_th_ (left y-axis) and ISPP slope (right y-axis) for ferroelectric transistors (b) without a mid-IL, (c) with SiO_2_ mid-IL, and (d) with TiO_2_ mid-IL, respectively.


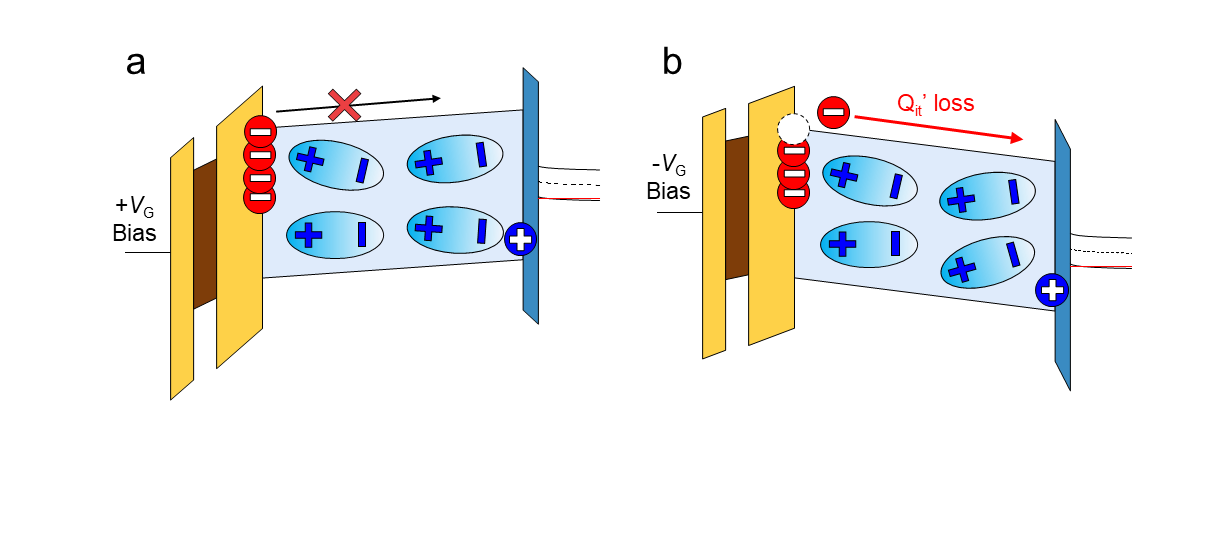


Supplementary Figure 9. Energy band diagrams illustrating gate-biased retention behavior. Band diagrams of MIFIS ferroelectric transistors without mid-IL in the erased state under (a) positive gate bias and (b) negative gate bias conditions, highlighting the mechanisms of gate-injected *Q*_it_’ loss.


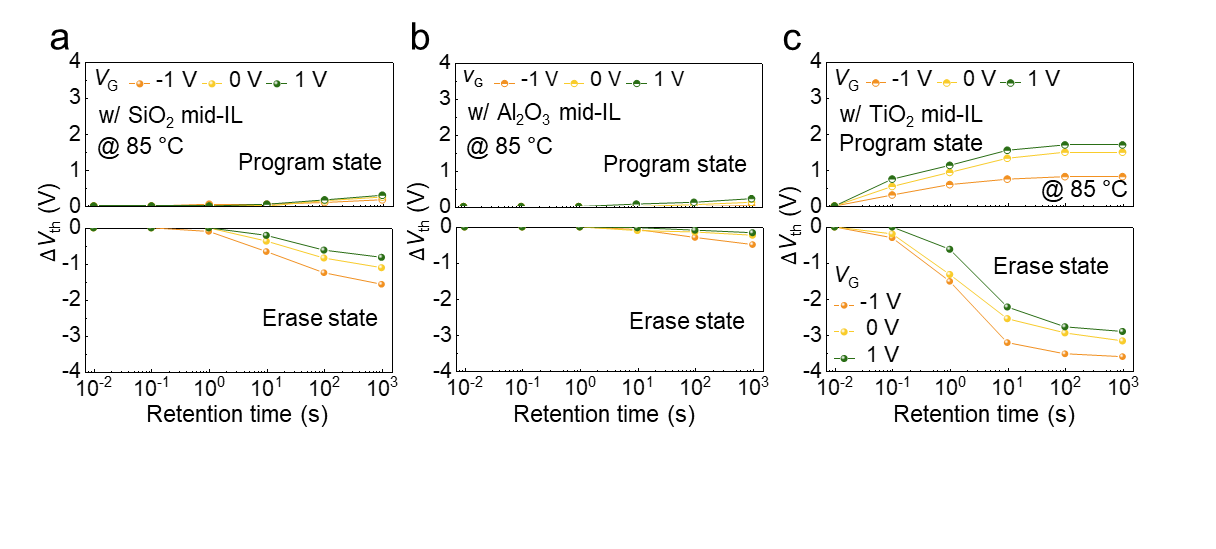


Supplementary Figure 10. Gate-biased retention behavior in ferroelectric transistors with different mid-ILs. Gate-biased retention characteristics of Fe-NAND cells with (a) SiO_2_, (b) Al_2_O_3_, and (c) TiO_2_ mid-ILs. Retention degradation is more pronounced under negative gate bias in the ERS state and positive gate bias in the PGM state, suggesting that gate-injected *Q*_it_’ loss toward the gate-side is the primary origin of charge loss during retention.


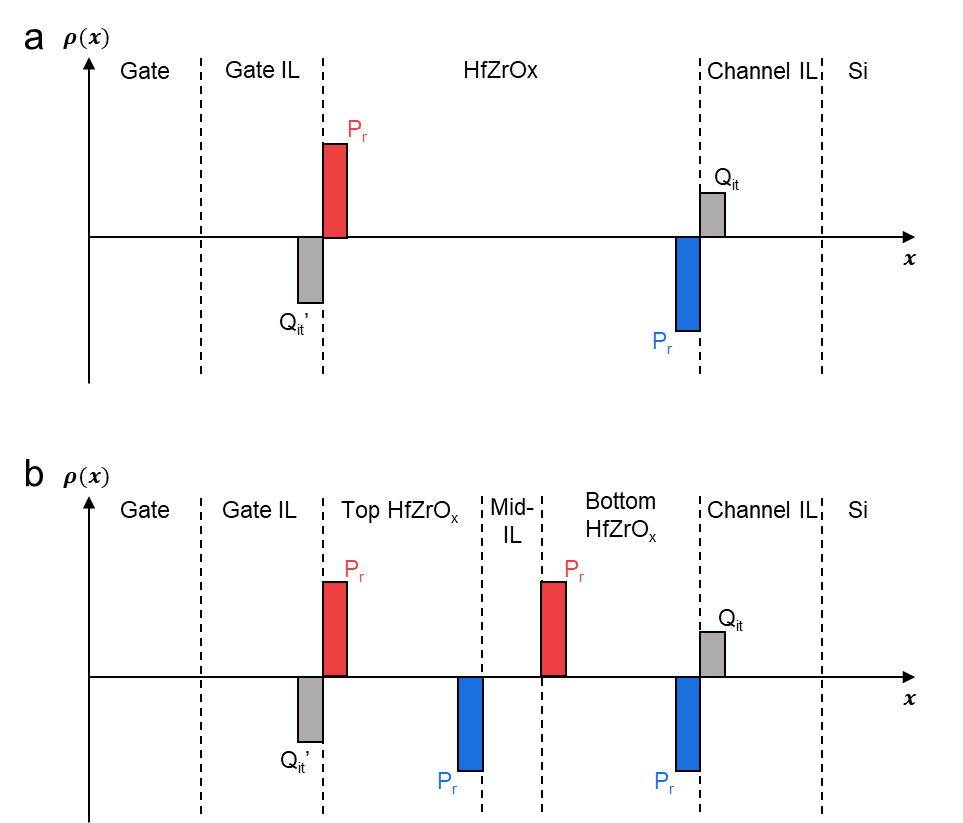


Supplementary Figure 11. Charge-sheet modeling to analyze the impact of mid-IL on retention. Charge distribution schematics of MIFIS gate stacks under retention conditions (VG = 0 V) in the ERS state: (a) without mid-IL and (b) with a mid-IL. Refer to Supplementary Text 2 for additional details.


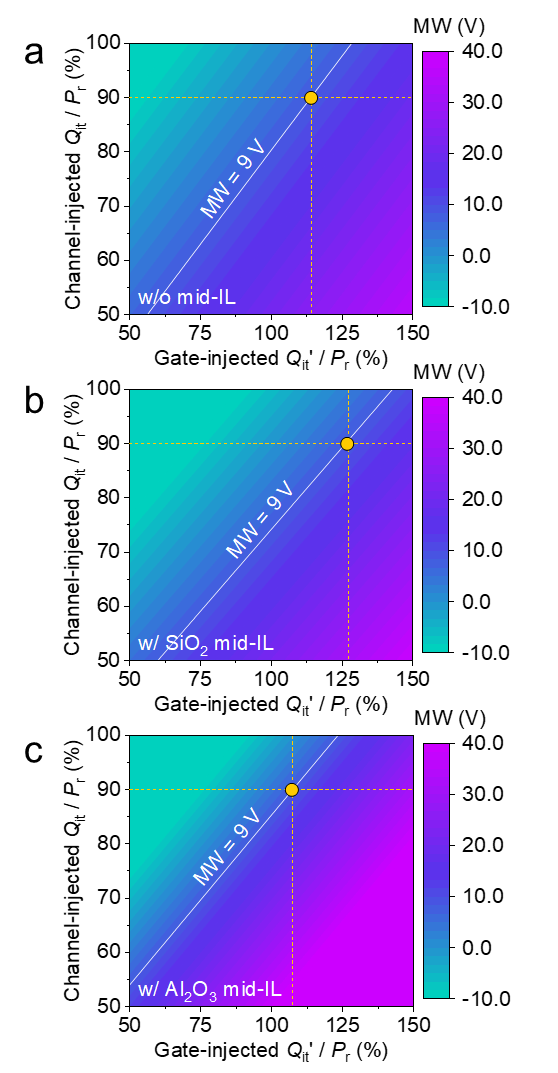


Supplementary Figure 12. Quantitative modeling of gate-injected *Q*_it_’. Contour plots of MW as a function of (gate-injected *Q*_it_’)/*P*_r_ and (channel-injected *Q*_it_)/*P*_r_ in ferroelectric transistors (a) without mid-IL, (b) with SiO_2_ mid-IL, and (c) with Al_2_O_3_ mid-IL. The coupling ratio between channel-injected *Q*_it_ and *P*_r_ is fixed at 90%. The conditions required to achieve an MW of ~9 V are marked by yellow circle symbols.


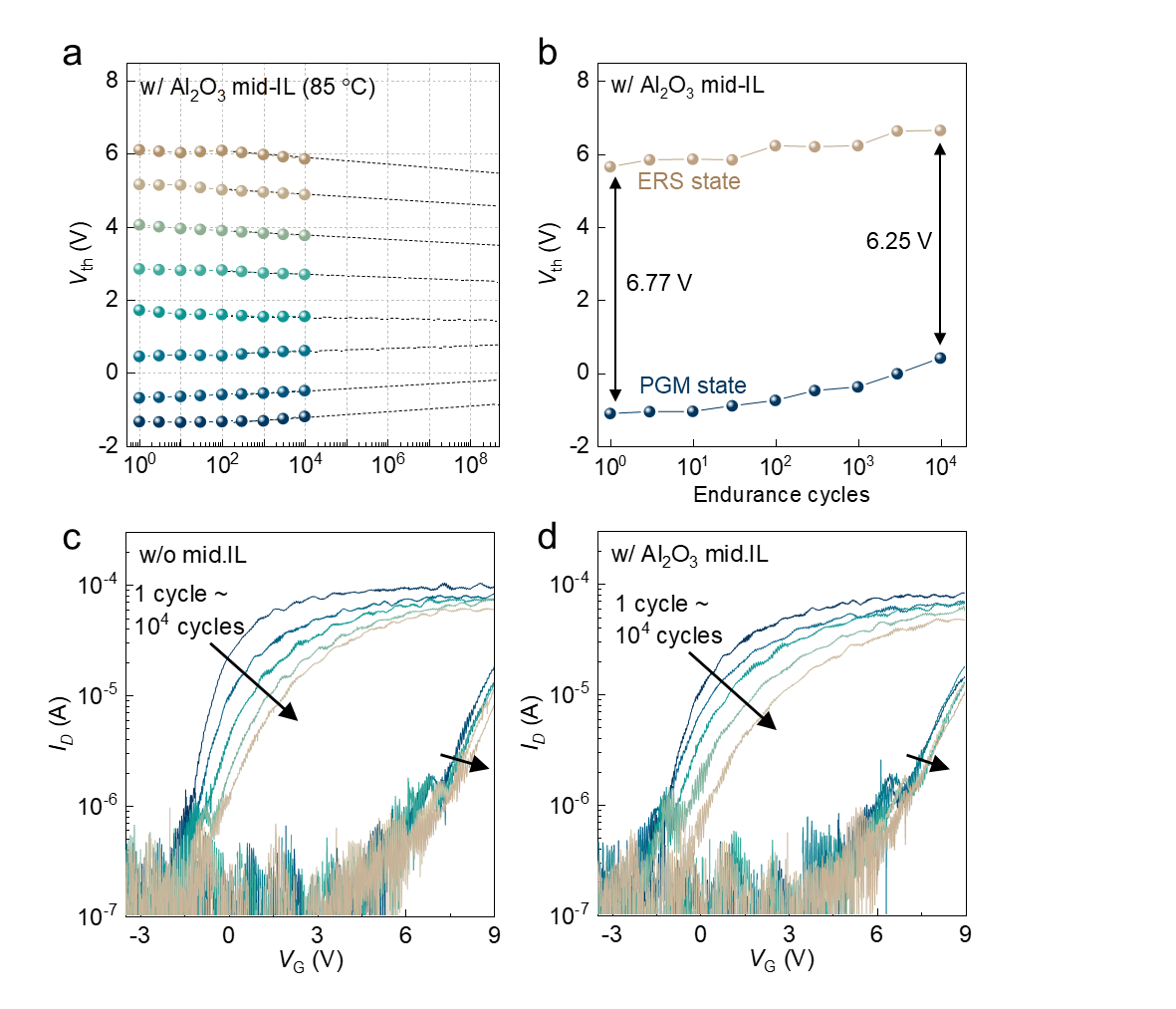


Supplementary Figure 13. Reliability of ferroelectric transistor with an Al_2_O_3_ mid-IL. (a) Triple-level-cell (TLC) retention characteristics of ferroelectric transistor incorporating an Al_2_O_3_ mid-IL at 85℃, confirming stable multilevel states over extended retention times. (b) Endurance behavior of the ferroelectric FET (FeFET) under repeated program/erase cycling, showing minimal degradation up to 10^4^ cycles. Evolution of pulse *I*_D_-*V*_G_ characteristics in MIFIS ferroelectric transistors (c) without and (d) with an Al_2_O_3_ mid-IL. These results highlight the robustness of the proposed gate stack engineering with mid-IL for reliable TLC operational FeFETs.


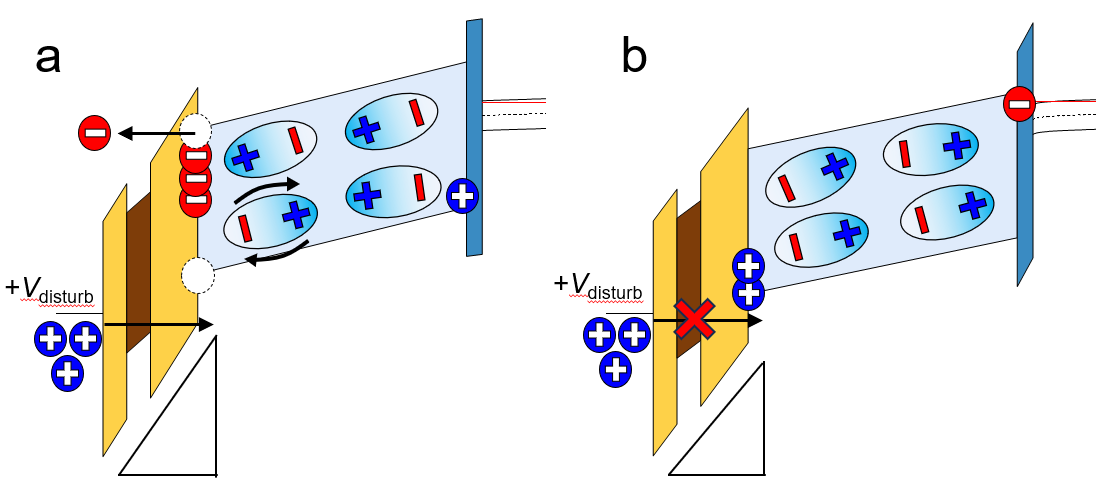


Supplementary Figure 14. Energy band diagrams of gate-injection-type ferroelectric transistors under disturbance conditions. The band diagrams of a MIFIS ferroelectric transistor under positive disturb bias applied to the gate (*V*_disturb_) in the (a) ERS state and (b) PGM state.


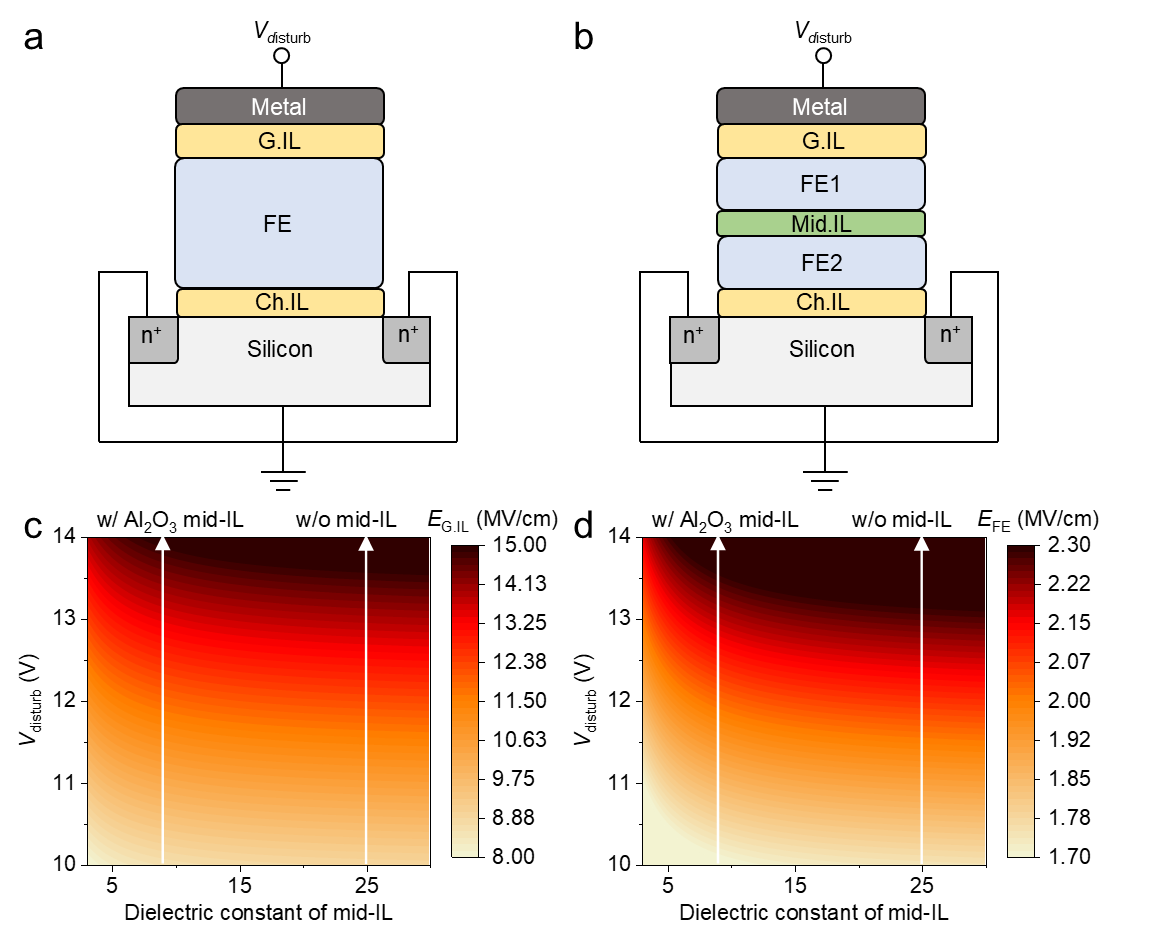


Supplementary Figure 15. Modeling analysis of mid-IL impact on disturbance in FeFETs. Schematic illustrations of MIFIS gate stacks under disturb voltage (*V*_disturb_) bias for (a) a conventional structure without mid-IL and (b) a Al_2_O_3_ mid-IL integrated structure. (c) Simulated electric field across the gate interlayer (*E*_G.IL_) and (d) ferroelectric layer (*E*_FE_) as a function of the mid-IL dielectric constant and applied *V*_disturb_ voltage. The results highlight how mid-IL integration, particularly with lower-κ materials compared to HfZrO_x_ (κ = 25), mitigates excessive electric field stress on both the gate interlayer and ferroelectric layer during disturbance conditions.


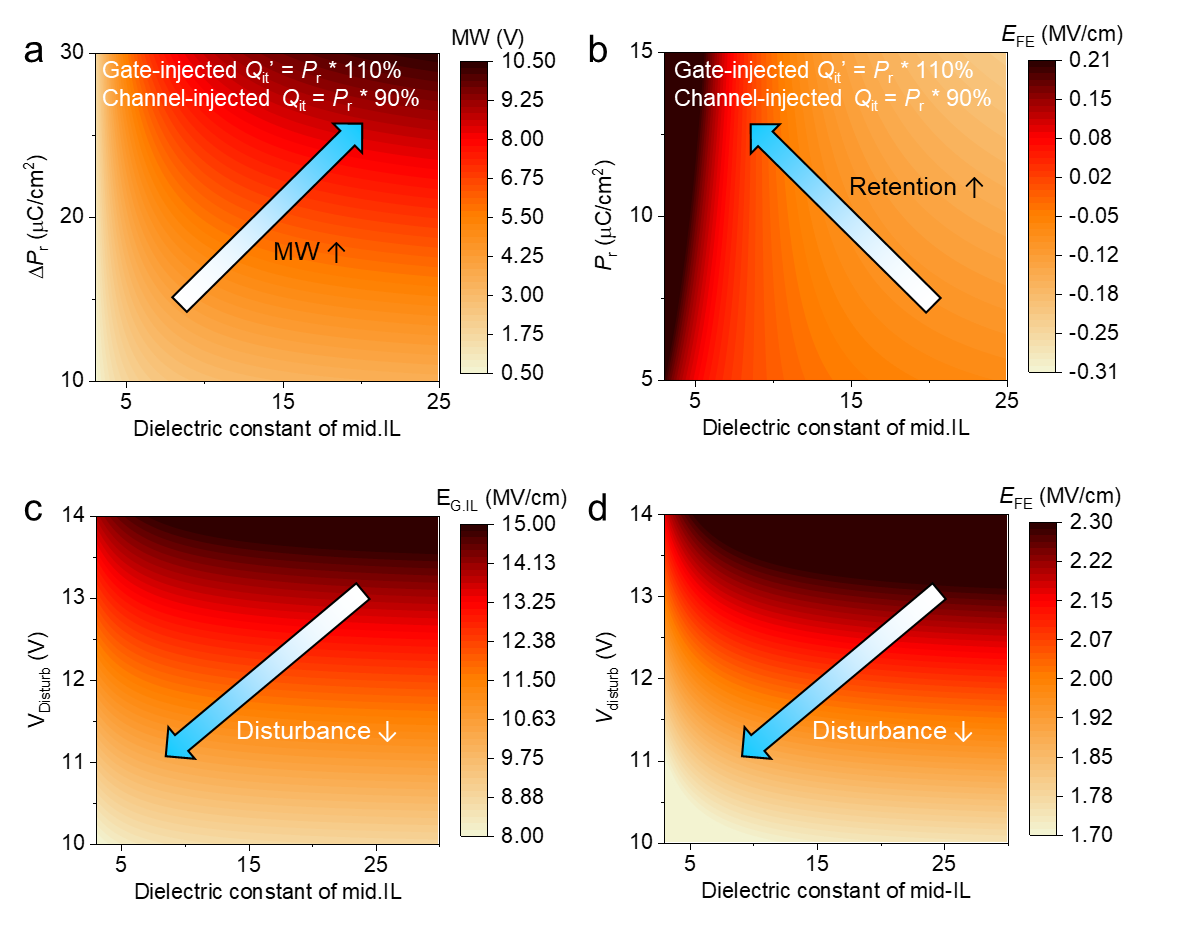


Supplementary Figure 16. Comprehensive modeling insights into mid-IL optimization for enhanced ferroelectric NAND (Fe-NAND) flash. (a) Contour plot of MW as a function of $\Delta$*P*_r_ and the dielectric constant of the mid-IL, showing enhanced MW characteristics with lower-κ mid-ILs. (b) Simulated *E*_FE_ during retention, plotted against *P*_r_ and mid-IL dielectric constant, highlighting the retention improvement with lower-κ mid-ILs. To facilitate comparative analysis, channel-injected *Q*_it_ and gate-injected *Q*_it_’ are assumed to be 90% and 110% of *P*_r_, respectively. Contour plot of (c) *E*_G.IL_ and (d) *E*_FE_ under disturb conditions, as functions of mid-IL dielectric constant and *V*_disturb_ amplitude. These results collectively suggest that optimizing the dielectric constant of mid-IL enables simultaneous enhancement of MW, retention and disturbance immunity.


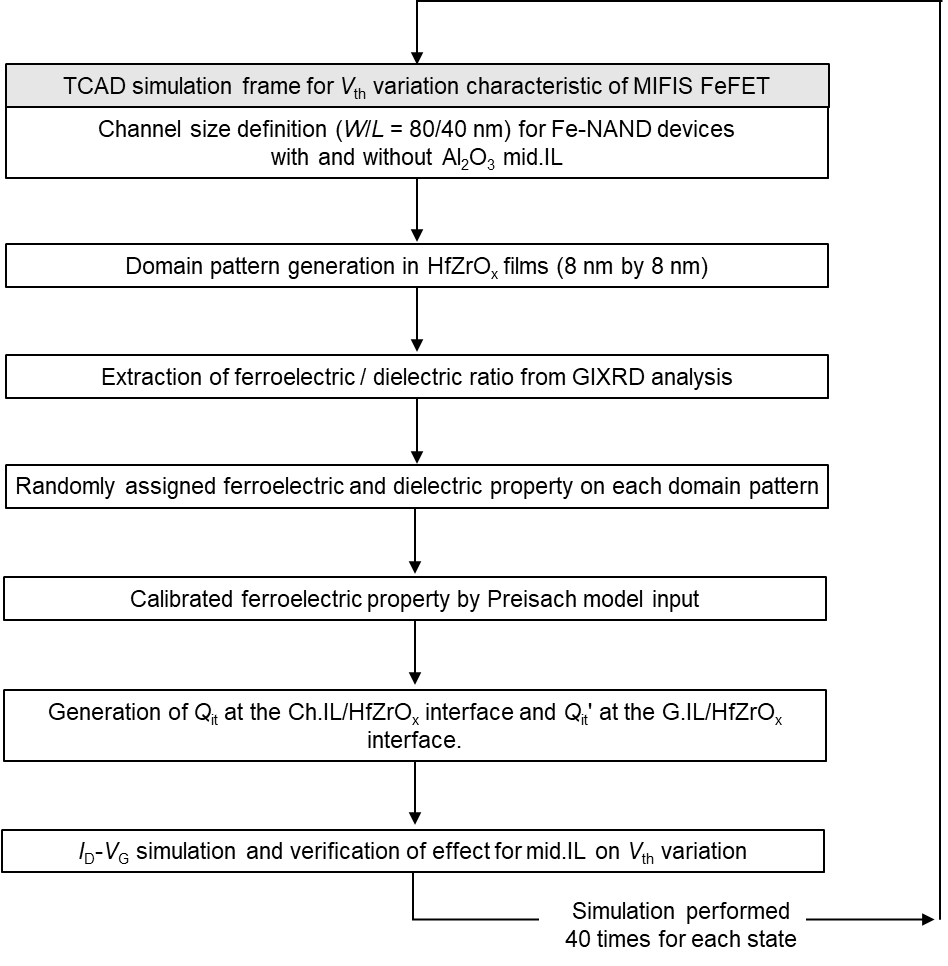


Supplementary Figure 17. TCAD simulation framework for analyzing *V*_th_ variability in FeFETs. The channel region of the ferroelectric transistors is defined with dimensions of 80 nm width and 40 nm length. An 8 nm $\times$ 8 nm domain pattern is introduced within the ferroelectric layers, where the spatial distribution of switchable and non-switchable domains is randomized based on the ferroelectric-to-dielectric phase ratio extracted from GIXRD analysis. The polarization switching behavior is modeled using the Preisach model to reproduce the experimentally observed hysteresis characteristics. Gate-injected *Q*_it_’ and channel-injected *Q*_it_ are incorporated at the respective interfaces. The *V*_th_ are extracted from simulated *I*_D_-*V*_G_ characteristics, and the simulation is repeated 40 times to statistically evaluate *V*_th_ variability.

Supplementary Table 1. Performance comparison of the state-of-the-art Fe-NAND flash devices. RT: room temperature.


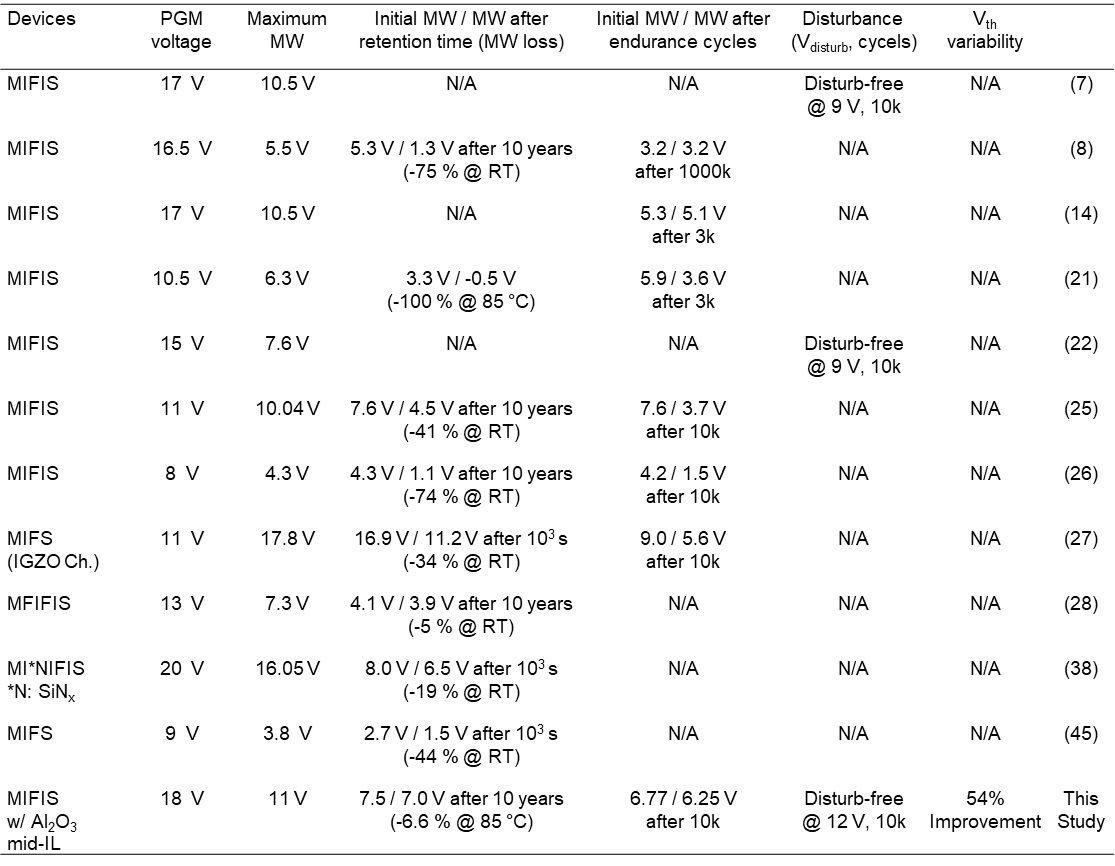

Supplement: Supplementary file 1 — Supporting Information [file ADVS-12-e10155-s001.docx]
